# The emerging role of chromatin remodelers in neurodevelopmental disorders: a developmental perspective

**DOI:** 10.1007/s00018-020-03714-5

**Published:** 2020-12-02

**Authors:** Britt Mossink, Moritz Negwer, Dirk Schubert, Nael Nadif Kasri

**Affiliations:** 1 Department of Human Genetics, Radboudumc, Donders Institute for Brain, Cognition and Behaviour, Geert Grooteplein 10, P.O. Box 9101, 6500 HB Nijmegen, The Netherlands; 2 Department of Cognitive Neuroscience, Radboudumc, Donders Institute for Brain, Cognition and Behaviour, 6500 HB Nijmegen, The Netherlands

**Keywords:** Epigenetics, Transcriptional regulation, Neurodevelopment, Radial glia, Neural progenitor, Chromatin accessibility

## Abstract

Neurodevelopmental disorders (NDDs), including intellectual disability (ID) and autism spectrum disorders (ASD), are a large group of disorders in which early insults during brain development result in a wide and heterogeneous spectrum of clinical diagnoses. Mutations in genes coding for chromatin remodelers are overrepresented in NDD cohorts, pointing towards epigenetics as a convergent pathogenic pathway between these disorders. In this review we detail the role of NDD-associated chromatin remodelers during the developmental continuum of progenitor expansion, differentiation, cell-type specification, migration and maturation. We discuss how defects in chromatin remodelling during these early developmental time points compound over time and result in impaired brain circuit establishment. In particular, we focus on their role in the three largest cell populations: glutamatergic neurons, GABAergic neurons, and glia cells. An in-depth understanding of the spatiotemporal role of chromatin remodelers during neurodevelopment can contribute to the identification of molecular targets for treatment strategies.

## Introduction

A mature brain is the product of its development. Early developmental insults during the assembly of these neuronal circuits can severely impact how a person develops and behaves in their adult life. Across the ongoing developmental continuum of progenitor expansion, differentiation, cell-type specification, migration and maturation, early developmental insults will compound over time, leading to a circuit dysfunction.

Neurodevelopmental disorders (NDDs) pose such an example of disorders where early developmental insults from conception on result in a wide and heterogeneous spectrum of clinical diagnosis’s including intellectual disability (ID), autism spectrum disorder (ASD), attention deficit hyperactivity disorder (ADHD), schizophrenia (SCZ) and mood disorders (bipolar disorder (BD), major depressive disorder (MDD) [1]. These NDDs are often diagnosed during childhood, and overlap between diagnostic categories [2]. NDDs can be caused by both genetic and non-genetic sources. The most frequent non-genetic cause of NDDs is foetal alcohol syndrome disorder [3–5]. Additionally, the extreme genetic heterogeneity in NDDs is one of the major limiting factors in both diagnosis and treatment [6]. With the use of sophisticated diagnostic tools such as whole exome sequencing and whole genome sequencing, the number of genes and variants linked to the aetiology of NDDs is vastly increasing [7, 8]. By doing so, chromatin remodelling genes have been found enriched in large datasets of NDD patients, and thereby pointing towards epigenetics as a convergent pathogenic mechanism [8–13]. By altering the epigenetic state of genes or histones, chromatin remodelers play an integral part in the machinery that translate external signals into lasting changes in gene expression patterns [14]. Furthermore, chromatin remodelers are multifunctional proteins that can influence various processes across the developmental continuum, including neural progenitor generation and specification, cell-type differentiation and expansion, migration and circuit integration [15]. Hence, as a significant subset of NDDs are caused by a failure of chromatin remodelling, it is to be expected that deficient chromatin remodelling will have a compounding effect across the developmental continuum, ultimately causing circuit dysfunction in mature networks [16].

Several reviews have already focussed on the most recent findings regarding the epigenetic origin of NDDs [17–19], however comparatively little is known about the function of these chromatin remodelers during the different programs of progenitor expansion, cell-type specification, neuronal migration and circuit integration. Using examples from mouse models mimicking these NDDs by introducing mutations in chromatin remodelers (also called NDD-related chromatinopathies), this review will first discuss how altered chromatin remodelling affects the different processes of the ongoing developmental continuum from mouse embryonic day (E) 10–17. Here, we will specifically focus on the three most abundant cell types found in the neocortex: excitatory (glutamatergic) and inhibitory (GABAergic) neurons, as well as glia cells. Although various chromatin remodelers have been described to play a role at one of these developmental processes, we chose to elaborate only on specific well-studied examples that play a role at multiple of these developmental steps, stressing their importance in neurodevelopment (Table 1). Furthermore, we will only focus on chromatin remodeler proteins and protein complexes and thereby exclude chromatin remodelling by non-coding RNAs (for a good review the authors would like to refer the readers to [20–22]).

**Table 1:** NDD-Associated Chromatin Remodelers

Gene	Complexes	Chromatin modifier function	NDD	Clinical phenotype	OMIM #	Animal phenotype	Cellular phenotype	Reference #
*ACTL6A*	SWI/SNF, nBAF	Actin-related	Non-specific ID, BOD Syndrome	ID, distinct facial features, delayed skeletal maturation, short stature	113477	–	Impaired stem cell renewal	[253, 254, 463, 464]
*ACTL6B*	SWI/SNF, nBAF	Actin-related	ID, Early Infantile Epileptic Encephalopathy	Intellectual disability, ambulation deficits, severe language impairment, hypotonia, Rett-like stereotypies, and minor facial dysmorphisms Epilepsy, Infantile Encephalopathy	612458	Memory deficits, reduced LTP	Reduced Bdnf signaling at NAc synapses, reduced MAP2 expression, dendritic abnormalitites	[253, 254, 464–467]
*ARID1A*	SWI/SNF, BAF	ATPase subunit	Coffin-Siris syndrome	ID, hypotonia, feeding problems, characteristic facial features, hypertrichosis, sparse scalp hair, visual problems, lax joints, short fifth finger, and one or more underdeveloped nails, corpus callosum underdevelopment or absence, microcephaly	614607	Craniofacial deficits	Reduced pluripotency and self-renewal of embryonic stem cells, increased potential to differentiate towards dopaminergic neurons	[279, 285, 465, 468]
*ARID1B*	SWI/SNF, BAF	DNA binding	Coffin-Siris Syndrome	ID, hypotonia, feeding problems, characteristic facial features, hypertrichosis, sparse scalp hair, visual problems, lax joints, short fifth finger, and one or more underdeveloped nails, corpus callosum underdevelopment or absence, microcephaly	135900	Small stature, weak muscle tone, corpus callosum hypoplasia, abnormal social, vocal, and behavioral phenotypes. PV-cKO: social and emotional impairments, SST-cKO:stereotypies, learning and memory dysfunction	E/I imbalance, altered number of GABAergic neurons	[277, 278, 280, 282, 283, 323, 353, 354]
*ARID2*	SWI/SNF, BAF	ATPase subunit	Coffin-Siris syndrome	ID, hypotonia, feeding problems, characteristic facial features, hypertrichosis, sparse scalp hair, visual problems, lax joints, short fifth finger, and one or more underdeveloped nails, corpus callosum underdevelopment or absence, microcephaly	617808	–	–	[285]
*ARL14EP*	SETDB1/KAP1/MCAF1 repressor complex	H3K9 methyltransferase	WAGR Syndrome	ID, Wilms Tumor, Aniridia	194072	–	Projection neuron generation via *Sema6a promoter methylation*	[324, 325]
*ARX*		Homeobox protein	ID, XLAG	X-linked lissencephaly with ambiguous genitalia (XLAG), agenesis of the corpus callosum (ACC), early-onset intractable seizures (EIEE1) and severe psychomotor retardation	300382, 300215	–	De-repression of *Scn2a*, *Syn1* and *Bdnf* in prenatal *Arx*^*−/Y*^ brains	[438, 441, 469–472]
*ATRX*	DAXX complex, CTCF/Cohesin binding	ATP-dependent DNA translocase, Histone variant exchange	Alpha-thalassemia/mental retardation syndrome(ATR-X), X-linked mental retardation-hypotonic facies syndrome-1 (MRXFH1)	ATR-X: Severe psychomotor retardation, characteristic facial features, genital abnormalities, alpha-thalassemia, ocular defects. MRXFH1: Mental retardation, microcephaly, short stature, unusual facial appearance, hypotonia	300032, 301040, 309580	Hemizygote males: Embryonic lethal. Heterozygote female cKO: Impaired spatial, contextual fear and novel object recognition memory, stunted growth	Sex-specific repression of miR-137, synaptic defects, loss of retinal interneurons (amacrine and horizontal cells)	[95, 299–308, 473–476]
*AUTS2*	PRC complex	H2AUb1 K119	Autosomal dominant form of syndromic mental retardation	ID, ASD, microcephaly, short stature and cerebral palsy	615834	Defects in: motor skills, vocalisation following maternal separation, neurocognitive ability, exploration, recognition and associative memory and learning and memory formation	Impaired progenitor migration, increased in cell death during in vitro corticogenesis, premature neuronal differentiation, altered neuronal morphology	[123, 124, 477]
*CBP*		Acetylation of H3K9, H3K14 and H3K27	Rubinstein-Taybi syndrome	ID, postnatal growth deficiency, microcephaly, broad thumbs and halluces, and dysmorphic facial features	180849	Microcephaly, anxiety, reduced exploration and curiousity, brain structure abnormalities in the corpus callosum, hippocampus and olfactory bulb	Increased progenitor proliferation, reduced glutamatergic and gabaergic neuron generation, astrocytes and oligodendrocytes generation	[41, 68, 162–165, 319, 355–359, 446]
*CDK5RAP2*	–	Centrosomal protein	Autosomal recessive primary microcephaly-3 (MCPH3)	ID, Microcephaly	604804, 608201	Microcephaly with hypoplasia of cortex and hippocampus	Reduced neuroepithelial differentiation, fewer and smaller progenitor regions, and premature neuronal differentiation	[455, 478]
*CHD1*	–	ATPase subunit	Pilarowski-Bjornsson Syndrome	ID, developmental delay, ASD features, speech apraxia, mild dysmorphic features	602118, 617682	KO: Early embryonic lethal with gastrulation defects. Heterozygote: No effects	KO: Decreased NSC self-renewal, increased apoptosis	[197–200]
*CHD2*		ATP-dependent remodeler	Broad spectrum NDDs, Dravet Syndrome	Developmental delay, ID, ASD, epilepsy and behavioural problems, photosensitivity	615369	Aberrant cortical rhythmogenesis, memory deficits	Less proliferative RGCs, more differntiated IPs and Neurons, shift in excitarory / inhiobitory neuron production, E/I imbalance	[197, 201–207, 351, 352]
*CHD3*	NuRD complex	ATP-dependent remodeler	Snijders Blok–Campeau syndrome	ID, developmental delays, macrocephaly, impaired speech and language skills, and characteristic facial features	618205	–	Increased number of deep layer neurons at the expense of upper layer neurons	[97, 156, 196, 207–210]
*CHD4*	NuRD complex	ATP-dependent remodeler	CHD4-related syndrome	Macrocephaly, ID, hearing loss, ventriculomegaly, hypogonadism, palatal abnormalities and facial dysmorphisms that are diagnosed by Sifrim–Hitz–Weiss syndrome	617159	–	Reduced cortical thickness, reduced NPC proliferation, premature cell cycle exit, increased apoptosis of premature born neurons	[97, 137, 196, 207, 208, 211–213]
*CHD5*	NuRD complex	ATP-dependent remodeler	ASD	–	–	Abnormalities in socialization and communication, and deficits in behavioral measures of empathy	Reduced migration in cortical excitatory neurons	[9, 196, 207, 215–219]
*CHD6*	–	ATPase subunit	Hallerman-Streiff Syndrome	Craniofacial and dental dysmorphisms, eye malformations, hair and skin abnormalities, and short stature	602118, 234100	Ataxia, coordination problems	–	[196, 219–223, 479]
*CHD7*	–	ATP-dependent remodeler	CHARGE syndrome	Hypoplasia of olfactory bulb and cerebellum, agenesis of the corpus callosum, microcephaly and atrophy of the cerebral cortex, coloboma, heart defects, growth retardation, genital hypoplasia, and nose and ear abnormalities	214800	Hypoplasia of olfactory bulb, cerebral hypoplasia, defects in the development of telencephalic midline and reduction of the cortical thickness	Impaired proliferation and self-renewal of RGCs, reduced OPC and cerebral granular cell survival and differentiation	[224–230, 232, 233, 235]
*CHD8*	REST complex	ATP-dependent remodeler	ASD	Macrocephaly, rapid early postnatal growth, characteristic facial features, increased rates of gastrointestinal complaints and marked sleep dysfunction	615032	Macrocephaly, abnormal craniofacial features, and ASD like behaviour	Prematurely depletion of the progenitor pool, negative regulator of the Wnt–β-catenin signalling pathway. Impairs dendrite and axonal growth and branching of upper-layer callosal projection neurons, and resulted in delayed migration	[226, 237–241, 243–249, 251, 322, 439]
*CHD9*	–	–	–	–	616936	KO: No effects	–	[286, 480]
*CTCF*	Cohesin complex	3D Chromatin loop organizer	NDD mental retardation, autosomal dominant 21 (MRD21)	ID, microcephaly, growth retardation	604167	–	Embryonic cKO: Telencephalic structure defects. Adult cKO: clustered Protocadherin misexpression	[28, 104–106, 186–190]
*DAXX*	DAXX complex	Histone variant exchange	Alpha-thalassemia/mental retardation syndrome(ATR-X)	ATR-X: Severe psychomotor retardation, characteristic facial features, genital abnormalities, alpha-thalassemia, ocular defects	301040, 603186	–	Impaired activity-dependent Histone H3.3 loading in active neurons. Knockdown: Elevated Gad67 expression	[299–301, 481]
*DNMT1*	PRC complex	H3K27me3	Hereditary sensory neuropathy type IE (HSN1E)	Hereditary sensory autonomic neuropathy with dementia and hearing loss or cerebellar ataxia, deafness, and narcolepsy	614116, 604121	Transcriptional derepression, p53-activation, and partial cell growth defects	Precocious activation of a post-migratory genetic program in GABAergic neurons, increased appoptosis in POA	[79, 343, 363, 364, 401, 483]
*DNMT3A*		DNA methylation	Tatton-brown-rahman syndrome	ID, tall stature, macrocephaly characteristic facial features, atrial septal defects, seizures, umbilical hernia, and scoliosis	615879	Long bone lenght, enlarged body mass, Increased anxiety like behavior, reduced activity and exploration	Clustered protocadherin expression regulation in pyramidal cells + cerebellar Purkinje cells	[78, 483–486]
*DNMT3B*		DNA methylation	Hirschsprung disease, immunodeficiency-centromeric instability-facial anomalies syndrome-1	–	142623, 242860	–	Clustered protocadherin expression regulation in pyramidal cells + cerebellar Purkinje cells, accelerated expression of proneuronal genes	[78, 483–486]
*DPF1*	SWI/SNF, nBAF	Histone binding	–	–	601670	–	Postmitotic expression	[255, 256]
*DPF2*	SWI/SNF, BAF, PRC2	ATPase subunit	Coffin-Siris syndrome	ID, hypotonia, feeding problems, characteristic facial features, hypertrichosis, sparse scalp hair, visual problems, lax joints, short fifth finger, and one or more underdeveloped nails, corpus callosum underdevelopment or absence, microcephaly	618027	–	Reduced ESC self-renwal and increased apoptosis. Increased expression of neuron related genes in Dpf2-/- EBs	[284, 466]
*DPF3*	SWI/SNF, nBAF	Histone binding	–	–	601672	–	Postmitotic expression	[255, 256]
*EED*	PRC2	H3K9/K27 methylation reader	Cohen-Gibson Syndrome	ID, overgrowth of multiple tissues, macrocephaly, speech delay, poor motor skills	605984, 617561	cKO: Postnatal lethal	Disturbed laminar identity in cortex, Dentate Gyrus malformation	[130, 131, 466, 487, 488]
*EHMT1*	G9a-GLP comnplex	H3K9me1,2	Kleefstra Syndrome	Microcephaly, mild to severe ID, ASD, developmental delay, speech problems, hypotonia, characteristic facial features, epileptic seizures, heart defects and various behavioural difficulties	610253	Anxiety, immobility, impaired social interaction	NDMA-mediated overexcitability, cPcdh misregulation, PV critical period delayed	[154, 158, 159, 287, 375, 381, 401, 402, 425, 437, 489–498]
*EHMT2*	EHMT1/EHMT2 complex	H3K9me1/2	–	–	604599	KO: Embryonic lethal. cKO: decreased exploratory behavior, decreased sucrose preference, increased cocaine preference, obesity, altered locomotion. Heterozygote: No effects	cKO: increased dendritic spine plasticity in nucleus accumbens neurons, knockdown: decreased neurite sprouting	[158, 401, 425, 493, 499, 500]
*EZH1*	PRC2	H3K27me1/2/3	–	–	601674	–	Reduced PSD95 expression in hippocampal cultures	[125, 501]
*EZH2*	PRC complex	H3K27me1,2,3	Weaver syndrome	Overgrowth and macrocephaly, accelerated bone maturation, ASD, developmental delay and characteristic facial features	277590	Macrocephaly	Premature RGC differentiation, increased generation of lower‐layer neurons, decreased upper‐layer neuron production, precocious astrocyte generation and differentiation, altered neuronal polarization and radial neuronal migration	[125, 127–129, 138, 364, 403, 404, 466, 487, 501–503]
*HDAC1*	NuRD complex, DAXX complex	Histone deacetylase	–	–	601241	Astrocyte-specific cKO: Lethal early postnatal	Impaired neuronal differentiation, premature apoptosis, impaired lower layer neuron generation. Knockdown: elevated Gad67 expression	[174, 176, 481, 504]
*HDAC2*	HDACs, NuRD	Histone deacetylase	Cornelia de Lange Syndrome	Developmental delay, limb abnormalities, congenital heart defects, altered development of the reproductive system, growth retardation and characteristic craniofacial features	122470	Accelerated extinction of conditioned fear responses, accellerated learning in ASST test	Reduced proliferation and premature differentiation, abnormal cell death, decreased production of deep-layer neurons and increased production of superficial-layer neurons, defects in oligodendrocyte production	[174, 176, 177, 504]
*HDAC8*		SMC3 deacetylase	Cornelia de Lange Syndrome, Wilson-Turner syndrome	X-linked intellectual disabilityID, hypogonadism, gynaecomastia, truncal obesity, short stature and recognisable craniofacial manifestations resembling but not identical to Wilson-Turner syndrome	122470, 309585	Craniofacial features,	Reduced proliferation and differentiation of progenitors, increased apoptosis	[178–181, 183, 184, 505]
*HP1γ*	–	Heterochromatin formation	–	–	604477	–	Impaired axon/dendrite growth, impaired callosal projections	[148, 320]
*INO80A*	INO80 complex	ATPase subunit/Histone variant exchange	–	–	610169	Homozygous KO: Embryonic lethal. Heterozygotes/cKO: Microcephaly, Motor neuro deficits, premature death	Delayed DNA damage repair response, premature senescence	[289, 292, 294, 506]
*KANSL1*	NSL complex	H4K16ac	Koolen-de Vries Syndrome, 17q21.31 deletion syndrome	ID, distinctive facial features, friendly behaviour	610443	Altered weight, general activity, social behaviors, object recognition, and fear conditioning memory associated with craniofacial and brain structural changes	–	[167, 168]
*KANSL2*	NSL complex	H4K16ac	Severe ID	–	–	–	–	[8]
*KAT6A*	MOZ/MORF	Lysine Acetyltransferase	Syndromic ID (KAT6A Syndrome)	ID, craniofacial dysmorphism, ocular defects	616268	Craniofacial dysmorphism, body segment identity shift	Reduced H3K9ac at *Hox* gene loci	[347–350]
*KAT6B*	MOZ/MORF	Lysine Acetyltransferase	Genitopatellar Syndrome and Ohdo/SBBYS Syndrome	Hypoplasia/agenesis, urogenital anomalies, congenital flexion contractures of the large joints, microcephaly, agenesis of corpus callosum, and hydronephrosis	603736, 606170	–	Reduced generation of Excitatory + inhibitory neurons	[68, 344, 346]
*KAT8*	NSL complex	Lysine Acetyltransferase (H4K16) H4K16 propionylation	Syndromic ID	Brain abnormalities, epilepsy, global developmental delay, ID, facial dysmorphisms, variable language delay, and other developmental anomalies	–	Cerebral hypoplasia, postnatal growth retardation and preweaning lethality	Reduced progenitor proliferation, precocious neurogenesis	[8, 166]
*KDM5C*	REST complex	H3 Lysine 4 Demethylase	Claes-Jensen type of X-linked syndromic mental retardation (MRXSCJ)	Severe ID, epilepsy, short stature, hyperreflexia, aggressive behavior and microcephaly	300534	Small body size, aggressive behavior, and reduced social activity and learning	Malformation of dendritic arbors and spines along with misregulation of neurodevelopmental genes	[76, 438, 443, 471]
*KDM6A*	COMPASS complex	H3K4me3, H3K27me3/me2/me1	Kabuki Syndrome	Moderate ID, postnatal growth retardation, dysmorphic facial features (long palpebral fissures, with eversion of the lateral third of lower eyelids, high arched eyebrows, long lashes, broad and depressed nasal tip, large ears), clinodactyly, and recurrent otitis media in infancy	300128	Drosophila: rough eyes, dysmorphic wings and modification of the sex combs	Regulates posterior HOX gene expression	[507, 508]
*KMD1A*	NuRD complex	H3K4me1/2 demethylase	CRPF Syndrome	Cleft Palate, Psychomotor Retardation, Distinctive Facial Features, Hypotonia	609132, 616728	–	Reduced NSC proliferation, inhibition: Reduced adult neurogenesis in DG	[509, 510]
*KMT2A*	MLL1/MLL complex	H3K4 methylation	Wiedemann-Steiner Syndrome	ID, Mental Retardation, distinctive facial appearance, hairy elbows, short stature, microcephaly,	605130	Increased anxiety, cognitive deficits	Loss of Immediate Early Gene expression, impaired synaptic short-term plasticity	[76, 443, 511, 512]
*KMT2C*	COMPASS complex	H3K4me1 and H3K4me3	Kleefstra Syndrome	ID, Language/Motor Delay, ASD	610253	Prenatal and postnatal growth retardation and lethality in some embryos	–	[437, 495]
*KMT2D*	ASCOM complex	H3K4 methylation	Kabuki Syndrome	Intellectual disability, facial and limb dysmorphic features, and postnatal growth retardation	147920	Craniofacial dysmorphism and cognitive deficit	–	[444, 508]
*MBD5*		DNA methylation reader	Kleefstra Syndrome, MBD5-associated neurodevelopmental disorder (MAND), 2q23.1 microdeletion syndrome	ID, Language/Motor Delay, ASD, distinctive craniofacial phenotype	156200	Small body size, abnormal social behavior, cognitive impairment, and motor and craniofacial abnormalities	Deficiency in neuronal outgrowth, altered E/I balance	[82, 83, 140, 437, 495, 513]
*MECP2*	NuRD complex	DNA methylation reader	Rett Syndrome	Arrested development between 6 and 18 months of age, regression of acquired skills, loss of speech, stereotypic movements (classically of the hands), microcephaly, seizures, and mental retardatio	312750	Altered sensory processing, impaired auditory learning,	Premature maturation of PV + cells including marker expression, morphology, and synaptic properties. Astrocytes significantly affect the development of wild type hippocampal neurons in a non-cell autonomous manner	[80, 81, 84, 140, 303, 371–376, 378, 380, 412–414, 514–516]
*NIPBL*	Cohesin complex	Cohesin regulator	Cornelia de Lange Syndrome	Developmental delay, limb abnormalities, congenital heart defects, altered development of the reproductive system, growth retardation and characteristic craniofacial features	608667	Repetitive Behaviour, Seizures	Small size, craniofacial anomalies, reduced brain size, hearing abnormalities, early postnatal mortality, dysregulated clustered Protocadherin expression	[193, 517]
*NR1I3*	–	Nuclear hormone receptor	Kleefstra Syndrome	ID, Language/Motor Delay, ASD	610253	Impaired memory function and increased anxiety	Increased cortical NEUN density and dispersion of hippocampal granule cells, increased NPY expression in CA1, altered astrocyte morphology and increased microglia body size in CA1	[495, 518]
*PHC1*	PRC complex	H2AUb1	Primary microcephaly-11 (MCPH11)	Significant reduction of brain size, particularly the cortex, in the absence of gross-structural defects, and variable degree of intellectual disability	615414	Cephalic neural crest defect, microcephaly, abnormal facies, parathyroid and thymic hypoplasia together with skeletal and cardiac abnormalities	Increased Geminin expression and causes several cellular defects. Via Geminin expression is thought to affect germinal differentiation, lineage commitment and early specification of neural cell fate	[122]
*PHF10*	SWI/SNF, BAF	Histone binding	–	–	613069	Embryonic lethal	Impaired stem cell renewal	[255, 256, 519]
*PHF6*	PAF1 transcription elongation complex, NURD complex	Transcription regulation	Borjeson-Forssman-Lehmann syndrome	Mild to severe mental retardation, hypogonadism, hypometabolism, obesity with marked gynecomastia, facial dysmorphism, narrow palpebral fissure, large and fleshy ears, tapered fingers	301900	–	Afffected neuronal migration towards upper layers, affects morphology of migrating progenitors	[520–522]
*PHF8*	–	Demethylate histone H3K9me2/me1, H4K20me1, and/or H3K27me2	Siderius-type X-linked syndromic mental retardation (MRXSSD)	–	300263	Deficits in long term potentiation, learning and memory	Affected axon guidance, regulation of neuronal gene expression, overactivation of mTOR signalling	[523, 524]
*PRDM8*	–	Histone Methyltransferase	Progressive myoclonic epilepsy-10 (EPM10	Progressive myoclonus epilepsy associated with Lafora bodies	616639	Elevated Scratching Behaviour	Impaired axonal targeting	[320, 321, 525]
*RAD21*	Cohesin complex	3D Chromatin loop organizer	Cornelia de Lange Syndrome (mild)	Mild ID, Growth retardation, minor skeletal anomalies, facial features that overlap findings in individuals with CdLS	606462	–	–	[192]
*RING1B*	PRC complex	H2A Ubiquitination	Syndromic ID	Microcephaly, impairment of additional language-, cognitive-, and adaptive social skills, ID, Schizophrenia	–	–	Prolonged the neurogenic phase and delayed the astrogenic phase in cultures of neocortical progenitors. Increased production of deep-layer neurons	[136, 139, 526]
*SETB2*		H3K9me3	ASD	–	–	Altered left–right symmetry, deficits in zebrafish gastrulation	Delayed mitosis and is essential for chromatin condensation and seggregation	[527–529]
*SETD5*	–	DNA methylation reader	SETD5 syndrome	Characteristic facial features, mild to moderate ID, delayed speech development, hypotonia, short stature, microcephaly, (febrile) seizures	615761	ASD-like behaviour, brain anatomical differences, reduced cortical thickness in L5/6	Altered neuronal morphology and hypoconnectivity, delayed network development,	[530, 531]
*SETDB1*	PRC complex	H3K9me3, DNA methylation	ASD, Schizophrenia, MDD, Prader-Willi syndrome	Prader-Willi syndrome: neonatal hypotonia, hypogonadism, small hands and feet, hyperphagia and obesity in adulthood	176270	Microcephaly	Loss of Super TAD, reduced number of layer V and VI basal progenitors, increased number of layer II and III neurons in the CP	[140–143, 145, 146, 150, 151, 402, 417]
*SMARCA2*	SWI/SNF, BAF	ATPase subunit	Coffin-Siris Syndrome, Nicolaides-Baraitser syndrome	Microcephaly,sparse scalp hair, distinct facial features, short stature, prominent finger joints, brachydactyly, epilepsy, moderate to severe ID and impaired language development	135900, 601358	Impaired social interaction and prepulse inhibition	–	[285, 423, 532]
*SMARCA4*	SWI/SNF, BAF	ATPase subunit	Coffin-Siris syndrome	ID, hypotonia, feeding problems, characteristic facial features, hypertrichosis, sparse scalp hair, visual problems, lax joints, short fifth finger, and one or more underdeveloped nails, corpus callosum underdevelopment or absence, microcephaly	614609	Malformed cerebral and cerebellar cortices, reduced midbrain and brainstem size	Premature neuronal differentiation and altered cortical layering, reduced glia differentiation	[252, 261, 279, 285, 419–423, 466, 468, 533–535]
*SMARCA5*	SWI/SNF, BAF	ATPase subunit	–	–	–	Partial agenesis of the corpus callosum	Reduced generation of upper-layer pyramidal neurons	[265–267, 271]
*SMARCB1*	SWI/SNF, BAF	ATPase subunit	Coffin-Siris syndrome, Kleefstra syndrome, DOORS syndrome	ID, hypotonia, feeding problems, characteristic facial features, hypertrichosis, sparse scalp hair, visual problems, lax joints, short fifth finger, and one or more underdeveloped nails, corpus callosum underdevelopment or absence, microcephaly,choroid plexus hyperplasia	614608, 220500, 610253	Small stature, reduced body weight, microcephaly, cerebellar midline defects, midline defects	Decreased progenitor proliferation, reduced number of total IPs and neurons, larger of choroid plexus mass	[277, 279, 282, 283, 285–287, 437, 495, 536]
*SMARCC1*	SWI/SNF, nBAF	ATPase subunit		Severe Neural Tube Defect (occipital encephalocele and myelomeningocele)	601732	Neural tube closure defect, Exencephaly	–	[257–260, 533, 537–539]
*SMARCC2*	SWI/SNF, BAF	ATPase subunit	ASD	ID, developmental delay, prominent speech impairment, hypotonia, feeding difficulties, behavioral abnormalities	618362	Impaired memory, reduced cortical size and thickness	Depletion of RGC like progenitors in the Dentate Gyrus, enhanced astrogenesis	[257–260, 533, 534, 538, 539]
*SMARCD1*	SWI/SNF, BAF	ATPase subunit	–	Developmental delay, intellectual disability, hypotonia, feeding difficulties, and small hands and feet	618779	Drosophila: defects in long-term memory	–	[540]
*SMARCE1*	SWI/SNF, BAF	ATPase subunit	Coffin-Siris syndrome	ID, hypotonia, feeding problems, characteristic facial features, hypertrichosis, sparse scalp hair, visual problems, lax joints, short fifth finger, and one or more underdeveloped nails, corpus callosum underdevelopment or absence, microcephaly	616938	–	Responsible for repression of neuronal genes via interaction with REST	[277, 279]
*SMC1A*	Cohesin complex	3D Chromatin loop organizer	Cornelia de Lange Syndrome (mild)	Mild-to-moderate ID and other CdLS symptoms	300040	–	–	[191]
*SMC3*	Cohesin complex	3D Chromatin loop organizer	Cornelia de Lange Syndrome (mild)	Mild-to-moderate ID and other CdLS symptoms	606062	Anxiety	Greater dendritic complexity and more immature synapses	[191, 541]
*SOX11*	–	Transcription factor	Coffin-Siris Syndrome	ID, Language Delay, poor motor skills, small head, scoliosis	600898	KO: Embryonic lethal	cKO:: Impaired NPC proliferation, neuronal migration, and differentiation	[256, 488, 542]
*SRCAP*	SRCAP complex	ATPase subunit/Histone variant exchange	Floating-Harbor Syndrome	Mild to moderate ID, short stature, typical facial features, delayed bone age, delayed speech development with normal general motor development	136140, 611421	–	Erroneous progenitor lineage commitment	[291, 294, 296]
*SUV39H1*	EHMT1/EHMT2 complex	H3K9me3	–	–	300254	–	–	[402, 528]
*SUZ12*	PRC2, EZH2-EED complex	H3K9/K27 methylation	Imagawa-Matsumoto Syndrome	Postnatal overgrowth, increased bifrontal diameter, large ears, round face, horizontal chin crease and skeletal anomalies	606245, 618768	KO: Early embryonic lethal. Heterozygotes: Neural tube defects, spinocerebellar abnormalities, hydrocephalus	–	[128, 502, 543]
*YY1*	INO80 complex, PRC	Transcription Factor, CTCF-binding	Gabriele-de Vries Syndrome	Cognitive Impairment, motor abnormalities, dysmorphic facial features	617557, 600013	Homozygote: Early embryonic lethal. Heterozygote: Neurulation defects, developmental retardation	Regulates GluR1 expression following depolarization	[106, 544–546]
*YY1AP1*	INO80 complex	Recruits YY1	Grange Syndrome	Borderline ID, hyptertension, bone fragmentation, Kidney malfunction, vascular disease	607860, 602531	–	–	[547]
*ZNF143*	Cohesin complex	3D Chromatin loop organizer	–	–	603433	–	–	[107]
*ZNF644*	EHMT1/EHMT2 complex	Transcription factor	Autosomal Dominant Myopia	Strong Myopia	614167, 614159	–	maintenance of proliferative identity and delayed formation of differentiated retinal neurons	[157, 548]
*ZNF711*		Transcription factor	ID	Non-syndromic ID accompanied by autistic features or mild facial dysmorphisms	300803, 314990	–	–	[442, 549, 550]

*ASD* Autism Spectrum Disorder, *ID* Intellectual Disability, *MDD* Major Depressive Disorder, *TAD* Topologically Accessible Domain, *KAT* Lysine Acetyltransferase, *HDAC* Histone Deacetylase, *KDM* Lysine Demethylase, *KMT* Lysine Methyltransferase, *HDM* Histone Demethylase, *DNMT* DNA Methyltransferase, *MOZ/MORF* Monocytic Leukemia Zinc Finger Protein/MOZ-related Factor, *NSL* Non-Specific Lethal, *NuRD* Nucleosome Remodelling and Deacetylase, *REST* RE1-Silencing Transcription Factor, *SWI/SNF* SWItch/Sucrose Non-Fermentable, *BAF* Brahma Associated Factors, *PRC* Polycomb Repressive Complex, *COMPASS* Complex Proteins Associated with Set1, *ASCOM* ASC-2 Complex​

## Chromatin remodelers

Chromatin was first described by Walther Flemming for the unique fibrous structures observed in cellular nuclei [23]. Chromatin is a highly dynamic structure that regulates the complex organization of the genome and thereby controls the gene expression underneath, and is composed of nucleosomes containing an octamer of histones (i.e. H2A, H2B, H3 and H4), wrapped by 147 base pairs of DNA and the linker histone H1 [24]. The distinction between condensed *heterochromatin* and open *euchromatin* structures were first reported by Emil Heitz [25], and can be altered by chromatin remodelers via three distinct mechanisms, including: (i) sliding of an octamer across the DNA (nucleosome sliding), (ii) changing the conformation of nucleosomal DNA, and (iii) altering the composition of the octamers (histone variant exchange). By doing so, chromatin remodelling facilitates downstream gene expression in a cell-type and cellular demand-specific way, stressing their important role during (neuro) development.

**Fig. 1:**
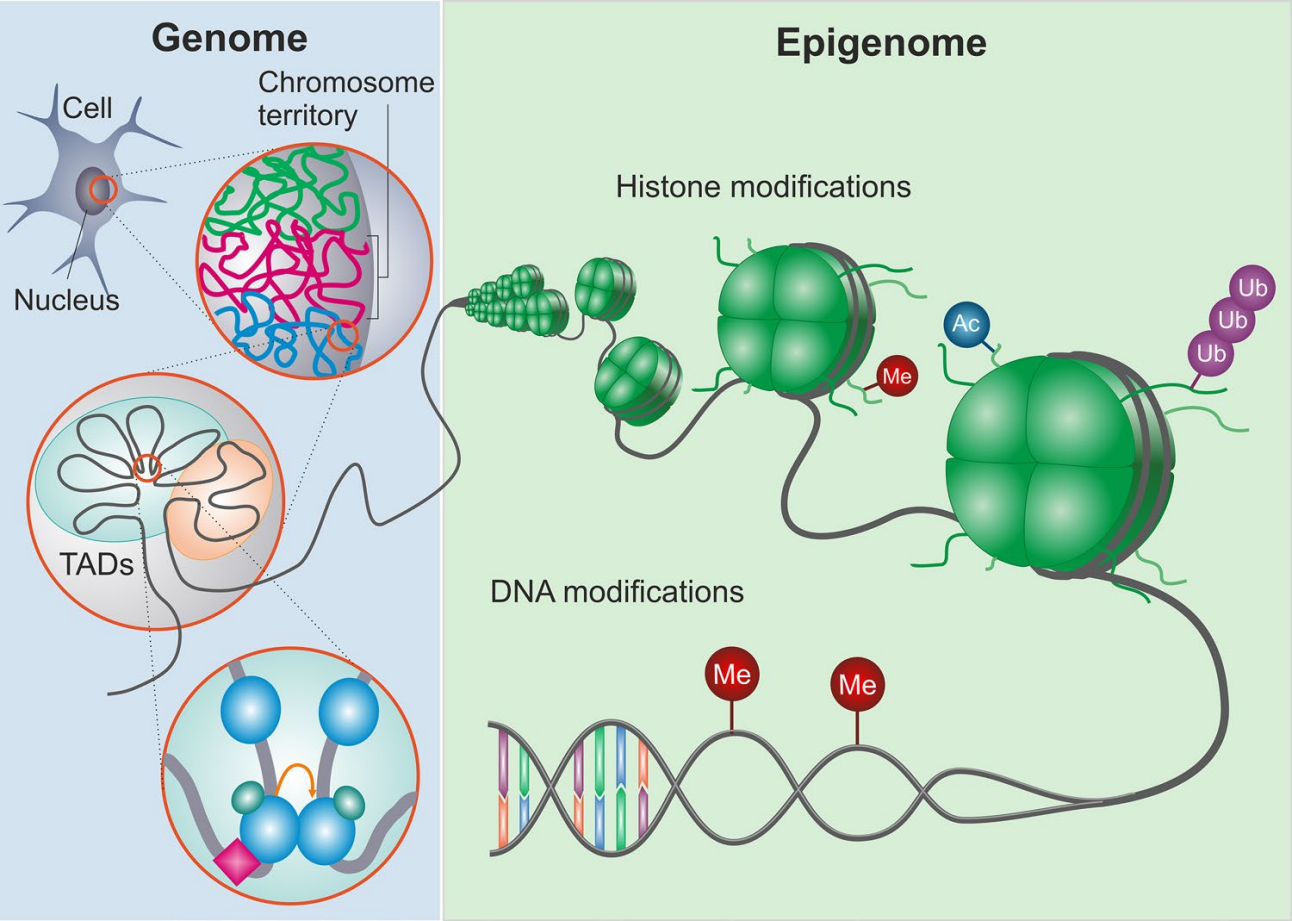
Left panel: The genome is organised into euchromatic (accessible) or heterochromatic (inaccessible) chromosome territories. Within chromosome territories, large chromatin domains are organised into smaller and smaller sub-domains known as topologically associated domains (TADs). TADs are regions where DNA is highly organised in 3D space to enable long-range transcriptional regulation between non-linearly neighbouring strands. Right panel: DNA is wrapped around an octamer of histones, of which its accessibility is regulated by histone modifications like methylation (Me), Acetylation (Ac) or Ubiquitination (Ub) or by histone modifying enzymes. Finally, transcription can be regulated by direct DNA modifications, such as DNA methylation

Based on their function, three categories of chromatin remodelers have been classified, which are (i) the enzymes that control histone post-translational modifications (PTM) [26], (ii) DNA modifications that can attract/repel chromatin remodelling proteins or complexes, or (iii) enzymes that alter histone-DNA contact within the nucleosome via ATP hydrolysis [27]. Furthermore, 3D genome architecture is increasingly considered an important epigenetic regulator of gene expression [28]. In the next section, we will briefly discuss the global function of these epigenetic regulators (Fig. 1).

### Histone modifying enzymes

Over the last decade a major effort was put into the identification of enzymes that directly modify histones. So far, enzymes have been identified for methylation [29], acetylation [30], phosphorylation [31], ubiquitination [32], sumoylation [33], biotinylation [34], ADP-ribosylation [35], deamination [36, 37], proline isomerization [38], β-N-glycosylation [39], crotonylation [40], propionylation [41], butyrylation [41], serotonylation [42], dopaminylation [43], Glutarylation [44–46], Lactylation [47], Benzoylation [48], S-palmitoylation [49], O-palmitoylation [50] and 5-Hydroxylysine [51]. Also nonenzymatic histone PTMs have been identified including Glycation [52, 53], 4-Oxononanoylation [54, 55], Acrolein adduct [56, 57], Homocysteinylation [58, 59] nitrosylation [60–63], sulfe‐, sulfi‐, and sulfonylation [64, 65] and S-glutathionylation [66, 67]. For many of them, also enzymes have been identified that can remove the PTM, or ‘read’ the PTM and recruit other proteins to form a chromatin remodelling complex (for a detailed review of all histone modifying enzymes and their function see [26, 68]). Currently, two mechanisms are known by which histone PTMs can alter the state of chromatin. First, it is accepted that all histone modifications have the potential to affect higher order chromatin structure by neutralising the basic charge of the nucleosome, and therefore could loosen inter or intra-nucleosomal DNA-histone interactions [69–72]. A well-known example is acetylation of lysine residues, which removes the positive charge of lysine and therefore increases the probability to alter the structural state of chromatin [73]. Second, histone PTMs can recruit non-histone proteins to set in motion processes such as transcription, DNA repair and DNA replication [73]. Depending on which histone modification (or which sequence of histone modifications) are present at a given histone tail, different sets of proteins are encouraged to bind or prevent from binding to chromatin. This recruitment process is highly dynamic, as multi-step processes (e.g. transcription) require a distinct set of histone PTMs to recruit a distinct set of chromatin-remodelling proteins. Proteins that are recruited to PTMs contain specific PTM-reader domains (such as bromo-domains, chromo-(like) domains, PhD domains etc.). Many chromatin remodelers actually possess multiple reader domains, suggesting their ability for multivalent interactions that would increase both affinity and specificity [74, 75]. Functional interplay among writer-eraser PTM enzymes in the brain remains largely unknown. Recent reports, however, showed that knockout mice of the writer-eraser duo *Kmt2a* and *Kdm5c*, which are responsible for Wiedemann-Steiner Syndrome and Mental Retardation X-linked Syndromic Claes-Jensen, share similar brain transcriptomes, cellular- and behavioural deficits [76]. Double mutation of *Kmt2a* and *Kdm5c* however partly corrected H3K4 transcriptomes as well as their cellular and behavioural deficits, suggesting this balance is essential during development and might be an interesting therapeutic strategy for NDDs [76].

### DNA methylation

In addition to methylation of histone tails, DNA can also be methylated to regulate chromatin state transitions (Fig. 1). The addition of a methyl group from S-adenosyl-L-methionine substrates only occurs on cytosines that are followed by guanines (called CpG sites), and is catalysed by DNA methyltransferases (DNMTs) leading to gene repression. There are two types of DNMT classes, namely either the de novo methyltransferases or maintenance methyltransferases [77]. DNMT3a and DNMT3b are classified as de novo methyltransferases as these can methylate previously unmethylated cytosine of CpG dinucleotides on both strands [78]. DNMT1 is classified as a maintenance methyltransferase as it has a substrate preference for hemimethylated DNA over unmethylated DNA. In contrast to its preference, DNMT1 can also display de novo methyltransferase activity in a specific cellular context-dependent manner [79]. DNA methylation can affect chromatin remodelling either by attracting transcriptional activators to the methylated cytosine [80], or it can attract transcriptional repressors that have methyl cytosine-binding domains. For example, DNA methylation can recruit histone deacetylases, which facilitate the formation of the silent chromatin state [81]. These methyl cytosine-binding proteins include methyl CpG-binding domains (MBDs) [82, 83] and methyl CpG-binding protein 2 (MeCP2) [84], which are known for their role in the aetiology of NDDs. During human development, genomic DNA methylation signatures are established in early development by two consecutive waves of nearly global demethylation, followed by targeted re-methylation [85, 86]. NDD mutations have often been found to underlie errors in methylation during these early time points [87, 88]. Consequently, altered methylation signatures during early development may be passed on across all cell lineages and can thus affect multiple tissues. When these epigenetic changes are maintained throughout development and across cell-types, these so called ‘episignatures’ can be used as biomarkers for the diagnosis of NDDs using easily accessible tissues such as peripheral blood [89–92]. Indeed, several very recent studies have already showed the potential for using disease-specific episignatures as diagnostic tool for NDDs, including patients with a known diagnosis as well as patients carrying variants of unknown significance [93–95].

### ATP-dependent chromatin remodelers

The third class of chromatin modifying enzymes are the ATP-dependent chromatin remodelers including SWI/SNF (switch/sucrose-non-fermenting), ISWI (imitation switch), CHD (chromodomain-helicase-DNA binding) and INO80 (inositol requiring 80). In general, ATP-dependent chromatin remodelers hydrolyse ATP to generate enough energy to disrupt the interactions between histones and DNA. By doing so, ISWI remodelers can alter nucleosome positioning to promote heterochromatin formation and thus transcriptional repression. The SNF2/SNF chromatin remodelling family act as DNA translocases, by destroying histone-DNA bonds forming a DNA loop that propagates around the nucleosome until it reaches the exit site on the other side of the nucleosome [14]. Furthermore INO80 chromatin remodelers in vivo have been shown to play a role in nucleosome eviction, and histone variant exchange of the histone dimer H2A-H2B by the H2AZ-H2B dimer [96]. Finally, CHD family members exert a heterogeneous set of biological properties. One of the best studied examples is the NURD (nucleosome remodelling and deacetylase) complex, which contains lysine‐specific histone demethylase 1A (LSD1), Chromodomain Helicase DNA Binding Protein 3 (CHD3) or CHD4, histone deacetylases (HDAC1 or HDAC2) and methyl CpG-binding domain (MBD) proteins. The NURD complex has been shown to deacetylate specific gene sets during development leading to transcriptional repression [97].

ATP-dependent chromatin remodelers all share a conserved core ATPase domain, however all ATP-dependent chromatin remodelers harbour exclusive domains adjacent to the ATPase domain [14]. Each of these domains play a role in the recruitment of remodelers to chromatin, interaction with specific histone modifications and/or are involved in the regulation of the ATPase activity of the remodelers (see [14] for a detailed review on their function).

### 3D chromatin architecture

3D genome architecture is increasingly considered as an important epigenetic regulator of gene expression. On a coarse level, genomes are organised into structures known as chromosome territories (Fig. 1) [98]. These chromosome territories separate euchromatic from heterochromatic regions, and are termed ‘A’ and ‘B’ compartments, respectively [99]. Within the chromosome territories, megabase-sized chromatin domains are organised into smaller and smaller sub-domains known as topologically associated domains (TADs) [100]. TADs can be found in either ‘A’ or ‘B’ compartments, and are separated by sharp boundaries across which contacts are relatively infrequent. Interestingly, the boundaries between TADs are strikingly consistent across cell divisions and between cell types, as roughly 50%–90% of TAD boundaries have been shown to overlap in a pairwise comparisons between cell types [100]. In ‘A’ compartments, TADs are regions where DNA is highly organised in 3D space to enable “long-range” transcriptional regulation. This long-range transcriptional regulation is possible because enhancers are in close physical proximity to the promoters of their target genes in 3D space, despite long stretches of intervening nucleotides [101]. This physical proximity allows protein complexes bound at enhancers to interact with those bound at promoters (i.e. called enhancer-promotor loops), thereby influencing transcription of target genes. CCCTC-binding factor (CTCF) and Cohesin are such proteins that facilitate chromatin looping interactions [102]. CTCF-mediated loop formation requires one CTCF at each end of the chromatin loop, which dimerize if they are facing each other in the opposite orientation [103]. CTCF interacts with Cohesin via its C-terminal tail [104] and may thus allow Cohesin to locate on a particular side of the interaction to anchor and stabilize the chromatin loop [105]. In addition to Cohesin and CTCF, other proteins such as YY1 [106], ZNF143 and Polycomb group proteins [107], repetitive elements and PTMs are enriched at TAD boundaries to support transcription. These repetitive elements at TAD boundaries have been found to act as specific anchor points to spatially organize chromosomes [108], whereas enrichment for the transcription marks H3K4me3 and H3K36me3 in TAD boundaries show an association with highly expressed regions and suggests that transcription itself plays a role in TAD organisation [109]. By doing so, 3D chromatin structures play an essential role in orchestrating the lineage-specific gene expression programs that underlie cellular identity [110].

In summary, the above examples show that cells possess a wide range of chromatin remodelers to translate external signalling cues into lasting changes in gene expression (Fig. 1). Interestingly, chromatin remodelers are especially strongly expressed in the brain [111], and it is therefore not surprising that impaired chromatin remodelling in any of the above described remodeler classes has been identified to a cause monogenic forms of NDDs [68, 112–114]. Chromatin remodelers are often multifunctional [15], and by doing so have the ability to play divergent roles in the multi-step continuum of brain development. This continuum encompasses neural progenitor generation and specification, cell-type differentiation and expansion, migration and circuit integration. Dysfunction of chromatin remodelers at any point during this developmental continuum will result in lasting changes on mature network function. In the next section we will discuss the different steps along this developmental continuum, and explain how altered chromatin remodelling at any of these steps ultimately affects the structure and function of mature neuronal networks.

## Epigenetic modulation during neurodevelopment and disease

**Fig. 2:**
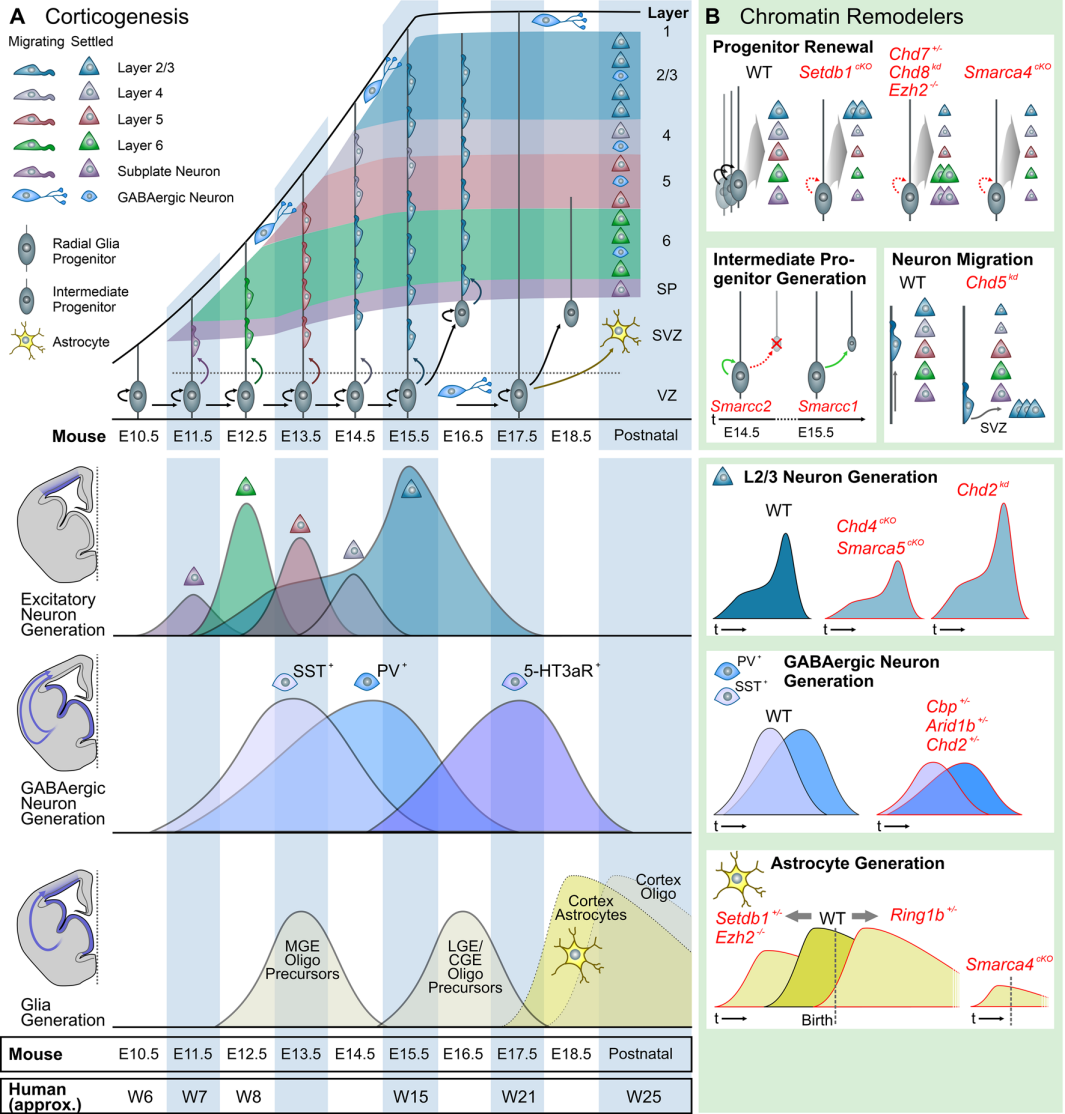
**a** Developmental timeline of the neocortex (top), with the generation times of the three most important cell classes indicated: Glutamatergic neuron generation in the cortical plate, glia production in the Medial Ganglionic Eminence (MGE) and cortical plate, and GABAergic neuron production in the Ganglionic Eminences (GEs). The timeline is based on mouse cortical development (indicated at the bottom as embryonal days (E), a mouse pregnancy lasts 19.5 days on average), with approximate human weeks post conception (W) displayed for reference. **b** The effect of chromatin remodelling defects on each cell population, with representative examples chosen from the NDD genes described in this review. Top four panels, defects in *CHD* family members (Chromodomain Helicase DNA Binding Protein), *Setdb1/Ezh2,* and BAF complex members on glutamatergic cell generation and maturation. Middle panel, comparable effects of mutations in two different chromatin remodelers on the generation of MGE-derived PV + and SST + neurons. Bottom panel, opposite effect of mutations in different chromatin remodelers on astrocyte generation timing

In the early stages of neocortical development, the telencephalic wall is composed neuroepithelial (NE) cells that will give rise to diverse pools of progenitor cells [115]. As these progenitors proliferate and expand in number, some begin to differentiate into radial glia cells (RGCs), establishing the ventricular zone (VZ). RGCs in turn begin to produce projection neurons and intermediate progenitors (IP) around E11.5 in mice which establish the subventricular zone (SVZ) [116]. In human development, RGCs not only produce projection neurons and IPs, but also the human specific outer radial glia (oRG) between gestational week (GW) 16–18, which will populate the outer SVZ (oSVZ) [117]. The RGCs in mice and oRG in humans act as transit-amplifying cells to increase the population of glutamatergic neurons until E18 in mice [118] or GW 21 in the human neocortex [119, 120], after which they switch to local glia production [121] (Fig. 2).

### Epigenetic modulation during neocortical development

#### Histone PTMs

Mutations in the Polycomb repressive complex (PRC) are a prime example of how defective chromatin remodelling influences progenitor proliferation. The PRC consists of two complexes: PRC1 and PRC2. Although for PRC1 no dominant germline mutations have been described, autosomal recessive mutations in the PRC1 complex protein PHC1 have been shown to cause a form of microcephaly with short stature in two Saudi siblings [122]. Loss of PHC1 resulted of the inability of patient cells to ubiquitinate H2A, resulting in increased *Geminin* expression causing cell cycle abnormalities and impaired DNA damage response [122]. Additionally, mutations of interactors of PRC1 are mutually vulnerable to cause NDDs with brain volume abnormalities. For example, mutations in the PRC1 interactor AUTS2 have been shown to cause an autosomal dominant form of syndromic mental retardation, including comorbidities such as ID, ASD, microcephaly, short stature and cerebral palsy [123, 124]. Mouse embryonic stem cells (mESCs) carrying heterozygous mutations in *Auts2* showed an increase in cell death during in vitro corticogenesis, which was rescued by overexpressing the human AUTS2 transcripts. Furthermore, mESCs harbouring a truncated AUTS2 protein (missing exons 12–20) showed premature neuronal differentiation, whereas cells overexpressing AUTS2 showed increase in expression of pluripotency markers and delayed differentiation.

The PRC2 complex consists of core subunits Enhancer of Zeste 2 (EZH2) and its homolog EZH1, which catalyse mono‐, di‐, and tri‐methylation of H3K27 resulting in heterochromatin formation and gene repression. *EZH2* is mainly expressed during embryogenesis, while *EZH1* is more ubiquitously expressed in adult and quiescent cells [125]. Loss-of-function mutations in *EZH2* and thus reduced H3K27me3 levels in humans have been shown to cause Weaver syndrome, causing overgrowth and macrocephaly, accelerated bone maturation, ASD, developmental delay and characteristic facial features [126–128]. In accordance with the overgrowth phenotype in Weaver syndrome, conditional KO of *Ezh2* in mice accelerated proliferation of neuronal precursors in the cerebral cortex at the expense of self-renewal of progenitors [129]. These results were strengthened by showing that fate-mapped E14-born neurons in a cKO for the PRC2 complex member *Eed* (which interacts with EZH2) mainly resided in the layer 2/3, in contrast to WT E14- born neurons, which resided in layer 4 [130]. These results confirmed PRC2 regulates the progression of apical progenitor’s temporal specification [130, 131].

Progenitors follow a specific pattern of cell divisions to initiate the build-up of the different layers in the cortex [132]. This is done in an ‘inside-out’ fashion, meaning the early-born neurons form the deep neocortical layers (i.e. 6 and 5), whereas late born neurons radially migrate through the deeper layers to create the more superficial layers (layer 4 and 2/3). Each RGC has been shown to consistently produce 8 to 9 glutamatergic neurons progressing from lower-layer excitatory neurons at mouse E12.5 to glutamatergic neurons that undergo radial migration towards the upper-layers at mouse embryonal day E15.5 [130]. Approximately 1 in 6 RGCs switches to gliogenesis, generating astrocytes and oligodendrocytes at the end of the cell cycle [133]. The process of neuroprogenitor specification during corticogenesis has been studied and reviewed in great detail elsewhere (see [134] or for a review see [116]) and will therefore not be discussed in this review. Numerous studies however have shown that defects in chromatin remodelers during RGC specification result in shifts in the neuron classes produced or in precocious cell cycle exit and gliogenesis, and thereby could contribute to the neuronal phenotypes found in NDD patients [135]. In mice, *Ezh2* is highly expressed in RGCs up to E14.5 and has been proposed to regulate RGC identity and proliferation behaviour, as well as RGC‐to‐glial‐progenitor transition [129, 136, 137] by inhibiting *Neurogenin 1* expression [136]. Ablation of *Ezh2* in mouse RGCs correlates with premature RGC differentiation as described earlier, increased generation of lower‐layer neurons, decreased upper‐layer neuron production, and precocious astrocyte generation [129]. Furthermore, knockdown of *Ezh2* in mice has been shown to affect the neuronal polarization and radial neuronal migration [138].

In addition to PRC2, deletion of the PRC1 component *Ring1b* at E13 prolongs the expression of *Fez transcription factor family member zinc-finger 2* (*Fezf2*). The prolonged expression of *Fezf2* results in the continuous expression of downstream target genes such as *Ctip2*, resulting in an increased production of deep-layer neurons [139]. Interestingly, when Ring1b is knocked out later in developmental time at E14.5, the number of upper-layer neurons is increased instead, due to an extended neurogenic period [136].

Similar to H3K27me, H3K9me2/3 is a repressive mark which is established by the methyltransferases SETDB1, (KMT1E), SUV39H1 (KMT1A), G9a (EHMT2, KMT1C) and G9a‐like protein (EHMT1, KMT1D). *SETDB1* has been associated to several NDDs. In humans, two missense mutations [140] and a microdeletion [141] in *SETDB1* were found in a large cohort of ASD patients. In addition, SETDB1 has been implicated to play a role in the aetiology of Schizophrenia by regulating *GRIN2B* expression [142]. Moreover, SETDB1 has been shown to influence chromatin 3D structure by binding to a non-coding element upstream of the *Pcdh* cluster [143, 144] which was a near-perfect match to a Schizophrenia risk haplotype (number 108 in [144]). Finally, SETDB1 is also described to play an indirect role in Prader-Willi syndrome, by contributing to the maternal silencing of the *SNORD116* gene [145, 146]. *Setdb1* is highly expressed early in corticogenesis (E9.5) in NE cells in the VZ, however its expression declines at E15.5 [135]. While deletion of *Setdb1* does not affect RGC numbers, it has been shown to reduce layer V and VI *Ctip2*^+^ basal progenitors between E14.5 and E16.5 [147]. At the same time, an increase of the number of *Brn2*^+^ layer II and III neurons was found in the CP. This shift in the production of upper layer neurons at the expense of deep layer neurons remained after neurogenesis ceased at E18.5. All these events together lead to a reduced cortical volume in *Setdb1* knockout mice at E18.5 and P7 [147]. Moreover, deletion of *Setdb1* causes accelerated astrogliogenesis, demonstrating that *Setdb1* does not only regulate the timing of late neurogenic events, but also the RGC‐to‐astrogenic‐progenitor transition [147]. As SETDB1 is a H3K9 methyltransferase, its general role is to repress gene transcription, or methylate DNA. SETDB1 does so by being involved in several complexes. SETDB1 interacts with The Krüppel-associated box (KRAB) domain-containing zinc-finger proteins (KRAB-Zfp) and KRAB domain-associated protein 1 (KAP-1) [148], which recruit the NuRD complex and HP1 to form a repressive complexes [149]. SETDB1 can also interact with MBD1 and ATF7IP [150], which has been suggested to play a role in X-inactivation [151]. To repress gene transcription via DNA methylation, SETDB1 interacts with DNMT3A/B in cancer cells, to represses the expression of *p53BP2* and *RASSF1A* [152]. Through the interaction with these complexes, SETDB1 can thus target various genomic loci in different cell types, at different stages during brain development.

Like SETDB1, EHMT1 plays a major role in embryonic development, as full knockout of this gene leads to embryonic lethality [153]. In hematopoietic stem cells, EHMT1 activity is essential to facilitate the long-term silencing of pluripotency genes, and thus inhibition of EHMT1 is essential for maintaining pluripotency [154]. Furthermore, a reduction of large H3K9me2 chromosome territories was found in these stem cells [154], which are proposed to stimulate lineage specification by affecting the higher order chromatin structure [155]. Little is known about the role of EHMT1 during early neurodevelopment, however knockdown of EHMT1 in NPCs revealed differential expression of genes important in development, such as BMP7, WNT7A, CTNNB1, TGFB2 and CHD3 [156]. Furthermore, in neural progenitors EHMT1 has been found to interact with ZNF644 to silence multipotency and proliferation genes. Disruption of the ZNF644/EHMT1 resulted in maintenance of proliferative identity and delayed formation of differentiated retinal neurons [157]. Similar roles for EHMT1 in the regulation neural progenitor genes were found in a conditional knockout mice (*Camk2a-Cre; GLP*^*fl/fl*^) [158]. Interestingly, no brain volume abnormalities are described in animal models of EHMT1 haploinsufficiency [158, 159] whereas microcephaly was found in 20% of patients carrying intragenic EHMT1 mutations [160]. These patients are diagnosed with Kleefstra Syndrome, which is in addition to microcephaly characterised by mild to severe ID, ASD, developmental delay, speech problems, hypotonia, characteristic facial features, epileptic seizures, heart defects and various behavioural difficulties [160, 161].

Histone acetylation by HATs and removal by HDACs is another example where deficits in chromatin remodelling affect the cellular distribution during corticogenesis. For example, the gene encoding the HAT cAMP‐response element binding protein (CBP) is highly expressed in proliferating RGCs and post‐mitotic neurons during corticogenesis [162], and CBP null mice have been shown embryonically lethal due to failure of neural tube closure (E9-E12.5) [163], stressing their role in neurodevelopment [164]. In humans, heterozygous mutations in *CBP* are associated with Rubinstein–Taybi syndrome (OMIM# 180849), which is characterized by ID, postnatal growth deficiency, microcephaly, broad thumbs and halluces, and dysmorphic facial features [68]. In mice, similar brain volume abnormalities have been described [165]. CBP induces acetylation of H3K9, H3K14 and H3K27 within target gene promoters, such as α*1‐tubulin* (E13‐E16), *Gfap* (E16‐P3), *S100β*, *Plp2* and *Mbp* (P14) [162]*.* Accordingly, heterozygous loss of *Cbp* in mice diminishes acetylation at these promoters and leads to decreased differentiation of progenitor towards astrocytes and neurons [162]. Consequently, a reduced number of neurons, astrocytes and oligodendrocytes were observed in the cortex of heterozygous *Cbp* mice around P14, whereas an increase in PAX6 expressing progenitors was found as compared to wild-type mice [162]. At later ages (P50), only a reduction in gliogenesis remained in the corpus callosum [162]. Another example of a HAT important in NPC development is Lysine acetyltransferase 8 (KAT8), a member of the Non-specific Lethal (NSL) complex which is responsible for acetylation of H4K16 and plays a role in H4K16 propionylation [166]. Cerebrum specific knockout of *Kat8* in mice has recently been shown to cause severe cerebral hypoplasia in the neocortex and in the hippocampus, together with postnatal growth retardation and pre-weaning lethality [166]. Furthermore, these mice showed a loss of RGC proliferation and thus reduced progenitor pool at E13.5, massive apoptosis starting at E12.5 and increased numbers of *Tuj1*^+^ cells, indicating precocious neurogenesis at E13.5 [166]. Similarly, patients with *KAT8* mutations presented with variable brain MRI abnormalities, epilepsy, global developmental delay, ID, facial dysmorphisms, variable language delay, and other developmental anomalies [166]. Interestingly, patients with epilepsy responded well to the histone deacetylase inhibitor Valproate [166], stressing the importance of KAT8 and other lysine acetyltransferases function in brain development.

Within the NSL complex, KAT8 is regulated by the NSL regulatory subunits KANSL1 and KANSL2. Mutations in *KANSL1* cause Koolen-de Vries Syndrome (OMIM# 610443), characterized by ID, distinctive facial features, and friendly demeanour [167]. Likewise, *KANSL2* mutations have been identified in ID patients [8]. Interestingly, haploinsufficiency of *Kansl1* in the mouse causes craniofacial abnormalities, reduced activity levels and impaired fear learning, as well as epigenetic dysregulation in genes linked to glutamatergic and GABAergic cells [168].

The balance between acetylation and deacetylation plays an important role in progenitor proliferation and differentiation, as inhibition of HDAC activity also results in progenitor proliferation/differentiation deficits [169]. Histone acetylation is removed by HDACs, resulting in chromatin condensation and transcriptional repression [170]. So far, over 18 HDACs are characterised in the mammalian genome, and they are expressed in a cell type- and developmental stage-dependent fashion [171]. For example, HDAC1 is highly expressed in neural stem cells/progenitors and glia, whereas HDAC2 expression is initiated in neural progenitors and is up-regulated in post-mitotic neuroblasts and neurons, but not in fully differentiated glia [172]. Conditional knockout of *Hdac1* or *Hdac2* in mice progenitors impairs neuronal differentiation [173]. Specifically, conditional knockout of *Hdac1* and *Hdac2* in *Gfap-Cre* mice resulted in major brain abnormalities and lethality at around P7 [173], whereas conditional knockout of *Hdac1* and *Hdac2* in *Nestin-Cre* mice resulted in reduced proliferation and premature differentiation of NPCs prior to abnormal cell death [174]. Moreover, inhibition of HDAC activity at the neurogenic stage decreases the production of deep-layer neurons and increases the production of superficial-layer neurons [175]. Conditional deletion of *Hdac1* and *Hdac2* in oligodendrocyte precursors results in severe defects in oligodendrocyte production and maturation [176]. Recently, the first patient carrying a mutation in *HDAC2* has been characterised presenting with many clinical features consistent with Cornelia de Lange Syndrome (CdLS) including severe developmental delay, limb abnormalities, congenital heart defects, altered development of the reproductive system, growth retardation and characteristic craniofacial features [177]. No patients harbouring mutations in HDAC1 have been characterized as to date.

Next to HDAC2, missense mutations in HDAC8 are also linked to the aetiology of CdLS presenting with overlapping clinical features (OMIM# 300269) [178, 179]. HDAC8 plays a key role in regulating cohesion function by deacetylating one of the core cohesion proteins, SMC3, which affects mitosis as well as transcription through loss of TAD function [180, 181]. Loss of HDAC8 activity in SVZ progenitors from 4-month old mice has been shown to reduce progenitor proliferation and differentiation [182]. Moreover, knockdown of HDAC8 in the mice embryonic carcinoma cell line P19 cells permitted the formation of embryoid bodies [183]. Furthermore, loss of HDAC8 in zebrafish has been found to increase apoptosis in CNS progenitors [182]. Recently a novel intronic variant in HDAC8 was found in a large Dutch family with seven affected males presenting with X-linked ID, hypogonadism, gynaecomastia, truncal obesity, short stature and recognisable craniofacial manifestations resembling but not identical to Wilson-Turner syndrome (OMIM# 309585) [184]. This variant disturbs the normal splicing of exon 2 resulting in exon skipping, and introduces a premature stop at the beginning of the HDAC catalytic domain [184]. How this specific variant influences neurodevelopment remains elusive.

#### Chromatin 3D organisation

The 3D organization of chromatin is changing dynamically as the cell differentiates. Whereas the nuclei of embryonic stem cells have been shown to be relatively homogenous, heterochromatin foci are becoming more apparent during differentiation into progenitors. When these progenitors differentiate into neurons, the heterochromatin foci are becoming even larger, suggesting that heterochromatin regions are actively reorganized during differentiation [185]. Deficiencies of 3D chromatin organizers such as CTCF and Cohesin are associated with the aetiology of NDDs called CTCF-associated NDDs and cohesinopathies, respectively. Heterozygous mutations in *CTCF* (OMIM# 604167) have been shown to cause the NDD mental retardation, autosomal dominant 21 (MRD21), which is characterised by variable levels of ID, microcephaly, and growth retardation [186–188]. In mice, loss-of-function studies of *Ctcf* revealed an important role for this protein in cell fate specification and neural differentiation. Knockout of *Ctcf* at E8.5 resulted in upregulation of PUMA (p53 upregulated modulator of apoptosis), leading to high levels of apoptosis and loss of the telencephalic structure [189]. Inactivation of CTCF several days later (E11) also resulted in PUMA upregulation and increased apoptotic cell death, and again the CTCF-null forebrain was hypocellular and disorganized at birth [189]. In contrast, conditional knockout of CTCF in postmitotic projection neurons resulted in misexpression of clustered protocadherin (*Pcdh*) genes leading to altered functional neuronal development and neuronal diversity [190]. These results suggest that CTCF activity regulates the survival of neuroprogenitor cells, and the balance between neuroprogenitor cell proliferation and differentiation [189].

The two best-described cohesinopathies are CdLS (OMIM# 122470, 300590, 610759, 614701, 300882) and Roberts syndrome (and its variant SC Phocomelia, OMIM# 268300). As described above, CdLS can be caused by loss of function mutations in HDAC2 and HDAC8. Additionally, mutations in three Cohesin subunits (*SMC1α, SMC3, RAD21*) [191, 192] and in one Cohesin-interacting protein (*NIPBL*) [193] have been found causal for CdLS, whereas mutations in *ESCO2* are responsible for Roberts syndrome/SC Phocomelia (OMIM# 269000) [194]. While a full knockout of Cohesin subunits in mice is lethal, mice carrying heterozygous mutations in these genes are viable and show altered gene expression in developmental programs, DNA repair and replication [195].

#### Chromatin remodelling complexes: CHD proteins and the NuRD complex

Altered chromatin remodelling by multi-subunit protein complexes has been shown to play a role in RGC differentiation and in the aetiology of NDDs. One of these complexes, called the NuRD complex, consists amongst other proteins of LSD1, HDAC1/2, and a Chromodomain Helicase DNA (CHD) Binding Protein (either CHD3, 4 or 5) [196]. These CHD proteins have been shown to play essential roles during neurodevelopment, as pathogenic variants in CHD1, CHD2, CHD3, CHD4, CHD6, CHD7 and CHD8 have been associated with a range of neurological phenotypes. Of the nine human CHD family members that have been characterized (CHD1-9), further subdivisions are being made into subgroups based on their function. Subfamily one consists of CHD1 and CHD2 because of their shared DNA binding domain that is not well-conserved in the other CHD proteins [197]. CHD1 has been found to play an essential role in early mice development, as C*hd1*^*−/−*^ embryos show proliferation defects and increased apoptosis, are smaller than controls by E5.5 and fail to become patterned or to gastrulate [198]. Similar results in decreased self-renewal and pluripotency were found using knockdown of *Chd1* in mESC cells [199]. Furthermore, *Chd1*^*−/−*^ ESCs show deficits in self-renewal and a reduction in genome-wide transcriptional output by directly affecting ribosomal RNA synthesis and ribosomal assembly [198]. In contrast, mice lacking a single *Chd1* allele (*Chd1*^+*/−*^) are healthy, fertile and phenotypically normal [198].

Recently the first patients with *CHD1* missense mutations were identified, which presented with ID, ASD, developmental delay, speech apraxia, seizures, and dysmorphic features. Interestingly, also a patient with a microdeletion spanning *RGMB* and the last exons of *CHD1* was characterised with no obvious NDD phenotype, suggesting that whereas deletions of *CHD1* may not cause a consistent neurological phenotype, missense mutations in *CHD1* may do so via a dominant negative mechanism [200].

Despite the fact that CHD family members are rather ubiquitously expressed, only *CHD2* pathogenic variants cause a brain-restricted phenotype, suggesting a unique role for this gene in neurodevelopment. Based on loss-of-function studies, CHD2 has been shown to regulate self‐amplification of RGCs and prevents precocious cell-cycle exit. CHD2 is mainly expressed in RGCs between E12-E18 and rarely in IPs, however knockdown of *Chd2 *in utero resulted in a reduction of RGCs in the SVZ whereas an increase was found in the number of produced IPs and neurons (Fig. 2) [201, 202]. This premature differentiation in RGCs can lead to a depletion of the progenitor pool, resulting in a smaller overall brain volume as a consequence [203]. Indeed patients harbouring mutations in *CHD2* present with a reduced head size and in 20% of the cases microcephaly [204, 205], developmental delay, ID, ASD, epilepsy and behavioural problems with phenotypic variability between individuals [206]. A subset of patients carrying CHD2 pathogenic variants present with developmental and epileptic encephalopathy (also called Dravet Syndrome), which is an early onset of epilepsy disorders characterized by refractory seizures and cognitive decline or regression associated with ongoing seizure activity [207].

CHD3, CHD4 and CHD5 are categorised in the second CHD family because they share dual plant homeodomain zinc finger domains [207]. Furthermore, these class 2 CHD remodelers exhibit subunit‐specific functions and display mutually exclusive occupancy within the NuRD complex at different stages of corticogenesis [208]. CHD3, has been shown to play an important role in the correct cortical layering and controls the timing of upper‐layer neuron specification [208]. In mice, CHD3 expression starts around E12.5 where it is still low expressed, and this increases from E15.5 to E18.5. At these later time points, CHD3 is mainly expressed in upper layer neurons in the cortical plate. Neurons lacking CHD3 (*CHD3*-knockdown) have been found more likely to express transcription factors that regulate laminar positioning and differentiation of deeper cortical layers (i.e. *Tbr1* and *Sox5*), whereas a lower number of neurons expressed the upper layer markers *Brn2* and *Cux1*, implicating that CHD3 may influence the expression of genes that couple radial migration with laminar identity (Fig. 2) [208]. Patients with CHD3 mutations are only very recently identified with Snijders Blok–Campeau syndrome, which is characterized by ID (with a wide range of severity), developmental delays, macrocephaly, impaired speech and language skills, and characteristic facial features [209, 210].

The second CHD protein that plays a role in the NuRD complex is CHD4. Mice lacking *Chd4* almost always died at birth, however when examining brain volumes at E18.5 a significant reduction of the cortical thickness was found, caused by reduced NPC proliferation and premature cell cycle exit, followed by increased apoptosis of premature born neurons [208]. As a result, *CHD4*^*fl/fl*^*/Nestin-Cre* mice presented with lower numbers of IPs and late born upper layer neurons (Fig. 2) [208]. Interestingly, *Chd4* appears to guide Polycomb repressor complex (PRC2) and especially *Ezh2* to opposing effects early vs. late in corticogenesis, first interacting to repress the gliogenic gene *Gfap*, and later repressing the neurogenic *Ngn1* after the neurogenic-to-gliogenic switch [137]. Interestingly, patients carrying mutations in *CHD4* actually present with macrocephaly, amongst other characteristics like ID, hearing loss, ventriculomegaly, hypogonadism, palatal abnormalities and facial dysmorphisms that are diagnosed by Sifrim–Hitz–Weiss syndrome [211]. The opposing phenotypes found for brain volume between rodent models and patients might be explained by a gene dosage effect, as for some variants in CHD4 altered ATPase activity levels were found, suggesting a possible gain-of-function phenotype in certain patients [212, 213]. Another possibility might be that the NuRD complex function is differentially regulated in humans and mice, as was recently suggested in a study comparing mouse and human pluripotent stem cells [214].

Finally, CHD5 has only recently been characterized as one of the core subunits of the NuRD complex [215]. CHD5 is the only CHD member that is mainly expressed in the total brain, foetal brain and cerebellum [216]. *Chd5* expression was mainly found in late-stage neuronal progenitors undergoing terminal differentiation, rather than in proliferating progenitors [215]. In utero knockdown of *Chd5* furthermore resulted in an accumulation of undifferentiated progenitors, which were unable to exit the VZ, SVZ and intermediate zone (IZ; Fig. 2) [215]. Additionally, knockdown of *Chd5* in mouse ESCs resulted in a failure to upregulate late stage neuronal genes [215]. Similar results for the role of *Chd5* in neuronal gene regulation were found in primary rat cultures [217]. A de novo damaging missense variant in the CHD5 gene was identified in an ASD proband from the Autism Sequencing Consortium [9]. In accordance, knockout of *Chd5* in mice indeed has been shown to cause ASD-like behaviour including increased anxiety and decreased social interaction [218].

The third subfamily consists of the remaining family members CHD6-9 [219]. CHD6 mutations have previously been described, including a large translocation in one Pitt-Hopkins patient [220], in a single case of mental retardation [221], in sporadic acute myeloid leukaemia incidences [222], and most recently for the very rare Hallermann–Streiff syndrome [223]. CHD6 is the least studied member of the CHD family, and little is known for its contribution during neurodevelopment.

Similar as to other members of the CHD family, loss of CHD7 resulted in impaired proliferation and self-renewal of RGCs in the SVZ. Consequently, a reduction of NE thickness in telencephalon and midbrain was shown in *Chd7* homozygous gene-trap mutant embryo at E10.5 [224]. Similarly, mESCs from *Chd7*^*Gt/Gt*^ mice showed a reduced potential to differentiate into neuronal and glial lineages, and presented with altered accessibility and expression of NPC genes. Furthermore, neurons generated from these *Chd7*^*Gt/Gt*^ mESCs presented with a significant lower length and complexity [225]. Furthermore, CHD7 plays a key role in oligodendrocyte precursor survival and differentiation (Fig. 2) [226, 227] and has been shown to cooperate with Sox10 to regulate myelination and re-myelination [227]. Additionally, CHD7 has been shown to play an important role in cerebral granular cell differentiation and cell survival [228].

Mice lacking one *Chd7* copy (found in *Chd7*^COA1/+^ mice [229] and *Chd7*^*Gt/*+^ mice [230]) also often present with brain abnormalities including the absence or hypoplasia of olfactory bulb, cerebral hypoplasia, defects in the development of telencephalic midline and reduction of the cortical thickness [229, 230]. Patients with *CHD7* mutations present with similar brain structure abnormalities including hypoplasia of olfactory bulb and cerebellum, agenesis of the corpus callosum, microcephaly and atrophy of the cerebral cortex [231–233]. Additionally, in relation to its role in oligodendrocyte differentiation and function, some patients have been characterised with white matter defects [234, 235]. Loss of CHD7 in patients is called CHARGE syndrome (OMIM# 214800), which is next to the brain malformations characterised by coloboma, heart defects, growth retardation, genital hypoplasia, and nose and ear abnormalities (including choanal atresia, deafness and vestibular disorders) [236].

Finally, CHD8 has been identified as a causal gene for ASD, presenting with common phenotypic features included macrocephaly, accompanied by rapid early postnatal growth, characteristic facial features, increased rates of gastrointestinal complaints and marked sleep dysfunction [237]. *Chd8* is strongly expressed around the transition from symmetric proliferative to asymmetric neurogenic RGC division [238], and knockdown of *Chd8* at E13 has been shown to prematurely deplete the neural progenitor pool in developing mice cortices (Fig. 2) [239]. Similarly, both heterozygous and homozygous knockout of *Chd8* in mESCs resulted in an upregulation of neuronal genes upon differentiation into NPCs [240]. Indeed, CHD8 binds the promoters of cell cycle genes and serves as a transcriptional activator of for example PRC2 components *Ezh2* and *Suppressor of Zeste 12* [239], which allows for the repression of neural genes during this developmental period [129]. In human iPSC-derived *CHD8*^+/−^ organoids, a number of ASD risk genes was upregulated [241]. Furthermore, CHD8 is identified as a negative regulator of the Wnt–β-catenin signalling pathway [242], as knockdown of *Chd8* in non-neuronal cells lead to an enrichment of up-regulated Wnt–β-catenin signalling pathway genes [239]. Interestingly, knockdown of *Chd8* in neuronal cells lead to an enrichment of down-regulated Wnt–β-catenin signalling pathway genes, indicating CHD8 plays cell-type specific roles [239]. Furthermore, conditional knockout of CHD8 in oligodendrocytes (*Chd8*^*flox/flox*^*;Olig1-Cre*^+*/*−^) has shown to impair oligodendrocyte differentiation and myelination in a cell-autonomous manner [243, 244]. Whereas homozygous deletion of *Chd8* in mice results in early embryonic lethality [245], *Chd8* heterozygous mice were viable and presented with similar phenotypes as patients, including macrocephaly, abnormal craniofacial features, and ASD like behaviour [246–250]. Moreover, introducing the human truncating variant N237K into the mouse *Chd8* gene (*Chd8*^+*/N2373K*^*)* revealed ASD-like behaviour, aberrant vocalization, enhanced mother attachment behaviour and enhanced isolation-induced self-grooming specifically in males, but not females [251]. These phenotypes were also conserved in zebrafish, where *Chd8* knockdown was found to cause macrocephaly and gastrointestinal phenotypes [237, 238].

#### Chromatin remodelling complexes: SWI/SNF

Also SWI/SNF chromatin remodelling complex subunits are expressed in a temporal and cell‐type specific manner [199, 252]. During differentiation from embryonic stem cells into neurons, the SWI/SNF complex begins to switch subunits to those unique to neural progenitors, followed by subunits specific to neurons [253, 254]. Neural progenitor proliferation requires a SWI/SNF complex containing PHF10 and ACTL6A subunit, which are replaced by the subunits DPF1, DPF3, and ACTL6B when neural progenitors exit the cell cycle to become post-mitotic neurons [255, 256]. Interestingly, the neural progenitor-specific SWI/SNF complex exclusively incorporates either SMARCC2 or SMARCC1 subunits at distinct developmental stages. In the mouse, neural progenitor SWI/SNF complexes harbours SMARCC2 until E14.5 to repress intermediate progenitor generation, whereas between E14.5 and E15.5, SMARCC2 is replaced by SMARCC1, activating intermediate progenitor generation in RGCs via the interaction with the H3K27 demethylases JMJD3 and UTX [257–259]. Double loss of *Smarcc2* and *Smarcc1* from as early as E10.5 (*Smarcc1*^*fl/fl*^*:Smarcc2*^*fl/fl*^*, Emx-Cre)* resulted in reduced numbers of proliferative progenitors, thinning of the cortical SVZ and loss of projection neurons [259]. In addition, loss of *Smarcc2* and *Smarcc1* in late NPCs (*Smarcc1*^*fl/fl*^*:Smarcc2*^*fl/fl*^*, hGFAP-Cre*) resulted in H3K27me3-mediated silencing of neuronal differentiation genes, causing delayed cortical and hippocampal neurogenesis [260]. Similarly, a loss of the catalytic subunit *Smarca4* in late NPCs (*Smarca4*^*fl/fl*^*::hGFAP-Cre*) resulted in reduced cortical thickness, dendritic abnormalities, hippocampal underdevelopment and cerebellar disorganization [261]. Furthermore, *Smarcc2* and *Smarcc1* double knockouts showed an upregulation of Wnt signalling activity resulting in increased progenitor proliferation-related defects [260]. This Wnt /β-catenin pathway has indeed previously been shown to be a critical regulator of NPC proliferation and neurogenesis during cortical development [116, 262, 263]. Thus, timely expression of these SWI/SNF subunits [264] is essential for regulating cell fate during neurodevelopment, and can control this processes by regulating Wnt signalling activity [262].

Not only SMARCC1 and SMARCC2 expression is essential during brain development, also divergent patterns of expression of SMARCA1 and SMARCA5 were found in the mouse embryo. Whereas *Smarca5* is mainly expressed in proliferating progenitors in the neocortex and the cerebellum, *Smarca1* is predominantly expressed in terminally differentiated neurons after birth in the cerebellum and hippocampus of adult animals [265, 266]. Consequently, *Smarca5*-null mice die during early preimplantation due to hypoproliferation of the inner cell mass [267], whereas *Smarca1*-null mice develop normally, but show hyperproliferation of progenitors causing an enlarged brain size [268]. Both remodelers have been shown to play a role in the proliferation and differentiation of IPs by controlling *FoxG1* expression. *FoxG1* is a critical transcription factor for IP proliferation and control of the timing of neurogenesis [269, 270]. On the one hand, conditional knockout of *Smarca5* in forebrain progenitors (*Emx*-cre) resulted in reduced *FoxG1* expression, impaired cell cycle kinetics and increased cell death. This resulted in a reduced number of *Tbr2*^+^ and *FoxG1*^+^ intermediate progenitors and thus a reduced cortical size [271]. Similarly, conditional knockout of *Smarca5* (*Nestin*-*cre*) caused reduced brain size and cerebral hypoplasia as a result of reduced granular progenitor proliferation [266]. On the other hand, loss of *Smarca1* (Ex6DEL) has been shown result in an increased *FoxG1* expression, a disruption of progenitor cell cycle kinetics, increased progenitor proliferation and increased neurogenesis [268]. Furthermore, *Smarca1* has been shown to directly bind to the promotor of the *FoxG1* gene, suggesting that timed chromatin remodelling by SMARCA1 is essential for controlling neuronal development and differentiation [268]. Taken together, these results confirm that timed chromatin remodelling of SWI/SNF-remodelers is essential during neurodevelopment [272]. It is therefore thus not surprising that misexpression of SWI/SNF complex subunits cause NDDs.

SWI/SNF-Related Intellectual Disability Disorders comprise a spectrum of disorders that includes the classic Coffin-Siris syndrome (CSS, OMIM# 135900) and Nicolaides–Baraitser syndrome (NCBRS, OMIM# 601358) [273]. These disorders differ amongst each other in a phenotypic spectrum ranging from syndromic ID over to classic and atypical/severe CSS to NCBRS. The core manifestations of CSS include ID, hypotonia, feeding problems, characteristic facial features, hypertrichosis, sparse scalp hair, visual problems, lax joints, short fifth finger, and one or more underdeveloped nails [274], and in a minority of the cases, microcephaly [275]. In contrast, NCBRS is defined by ID, short stature, microcephaly, typical face, sparse hair, brachydactyly, prominent interphalangeal joints, behavioural problems, and seizures [276]. Interestingly, structural brain midline defects such as corpus callosum malformations or even absence, are described in the majority of CSS patients [274, 277–280] and in some NCBRS patients [281], and animal models for these disorders [282].

The mild form of CSS is caused by either mutations in the ATPase subunit ARID1B (OMIM# 614556) [283], or by pathogenic changes in other chromatin remodelling proteins with no direct interaction with SWI/SNF complex, including SOX11 (OMIM# 600898) [256] and DPF2 (OMIM# 601671) [284]. Additionally, classic and more severe CSS are known to be caused by mutations in *SMARCA4* (OMIM# 603254), the common core subunit SMARCB1 (OMIM# 601607), and accessory subunits such as SMARCE1 (OMIM# 603111), ARID1A (OMIM# 603024) and ARID2 (OMIM# 609539) [285]. NCBRS on the contrary has been shown to be caused by mutations in *SMARCA2* (OMIM# 600014). Other patients found with mutations in SWI/SNF complex subunits are described for *SMARCB1* (OMIM# 601607) have been shown to cause either CSS, but also DOORS syndrome (OMIM# 220500) or Kleefstra syndrome (OMIM# 610253) depending on the site/location of the mutation [286, 287]. Together, these studies on chromatin remodelling complexes support the notion that combinatorial assembly of subunits can instruct cell lineage specification by creating specific patterns of chromatin states at different developmental stages, are essential for normal neurodevelopment [288, 289], and will result in clinical overlapping phenotypes [279].

#### Chromatin remodelling complexes: histone variant remodelers

The third class of ATP dependent chromatin remodelers is the INO80 family. The INO80 subfamily is known for its role in histone variant exchange of canonical H2A or H3 histone variants, which is assisted by editing remodelers such as Swr1 complex (SWR1C) [290], mammalian Snf2-related CBP activator protein (SRCAP) [291] and p400. Recently, INO80 function in NPCs has been found essential in homologous recombination (HR) DNA repair in a p53-dependent manner [292]. Loss of *Ino80* in NPCs (*Neurod6*^*Cre/*+^;*Ino80*^*fl/fl*^) impairs these processes, causing apoptosis and microcephaly in mice [292]. Interestingly, *Ino80* deletion in mice leads to unrepaired DNA breaks and apoptosis in symmetric NPC-NPC divisions, but not in asymmetric neurogenic divisions [292]. In correspondence with these findings, INO80 was recently identified as a candidate gene for ID and microcephaly [293]. Among the key histone variants that can be incorporated by the INO80 family is the H2A variant H2A.Z. Specifically, it has been shown that SRCAP removes canonical H2A–H2B dimers and replaces them with H2A.Z–H2B dimers [294]. Little is known yet how mutations in SRCAP affect neurodevelopment. However, mutations in SRCAP have been shown to cause the NDD Floating Harbour Syndrome (OMIM# 136140), which is characterized by intellectual and learning disabilities, a short stature, delayed osseous maturation, language deficits, and distinctive facial features [291, 295–298].

In addition to the INO80 family, another mechanism for histone exchange is suggested for the α-thalassemia X-linked mental retardation (ATRX) protein. ATRX is an ATP-dependent DNA translocase belonging to the Swi/Snf family of chromatin remodelers [299]. ATRX forms a complex with death domain associated protein (DAXX) [299, 300], and plays a critical role in the replication-independent deposition of the histone variant H3.3, functioning as a histone chaperone at specific genomic regions, including the telomeric domains [301, 302]. Furthermore, ATRX is involved in the suppression of several imprinted genes in the neonatal brain by promoting 3D chromatin structures via CTCF and cohesion [303]. In mice, germline deletion of *Atrx* has been shown embryonic lethal [304], whereas conditional deletion of *Atrx* in NPCs (*Foxg1*^*KiCre/*+^) caused a widespread cellular reduction in both the neocortex and hippocampus resulting in a significant smaller forebrain size [305]. In addition, *Atrx*^*Foxg1Cre*^ mice show excessive DNA damage caused by DNA replication stress and subsequent Tp53-dependent apoptosis [306, 307]. On a cellular level, these *Atrx*^*Foxg1Cre*^ mice show a reduction in precursor cell number and abnormal migration of progenitors in the hippocampus and the upper layers of the cortex [305, 306]. Furthermore, fewer Neuropeptide Y (NPY), SST and cholecystokinin (CCK) expressing GABAergic neurons were generated in the ventral telencephalon [306]. In humans, mutations in ATRX cause the rare congenital X-linked disorder ATRX syndrome (OMIM# 301040), characterised by moderate to severe ID, DD, microcephaly, hypomyelination, and a mild form of a-thalassemia [308].

To summarize, chromatin remodelers are highly expressed in neural progenitors, and are essential to dynamically activate, repress, or poise gene expression during the transition from RGCs to glutamatergic neurons or glia (Fig. 2, Table 1). Epigenetic modulation in glutamatergic neuron maturation is reviewed in detail elsewhere [135], however in the next section we want to highlight the maturation a specialized glutamatergic subpopulation that is frequently impacted in NDDs, called callosal projection neurons.

### Epigenetic modulation in callosal projection neuron development

The corpus callosum is a critical link between the two cortical hemispheres. The developmental mechanisms for callosal projections have been well researched in mice (reviewed in [309, 310]), and abnormalities of the corpus callosum often feature in human NDDs, especially ID and epilepsy [311] but also in Coffin-Siris Syndrome [312]. During mouse brain development, the cortical hemispheres fuse along the midline around E16 [313], aided by specialized glia populations called midline zipper glia and indusium griseum glia. Those establish the glial wedge to both sides, as well as the bridge-like subcallosal sling. Subsequently, callosal-projecting cortical pyramidal neurons start projecting axons across the midline and connect to their homotopic cortical area. Those projection neurons mostly reside in the upper cortical layers, and their callosal-projecting identity is under direct epigenetic control.

In newly generated pyramidal cell precursors, the transcription factor *Ctip2* specifies a subcortical-projecting fate, and is normally expressed in layer 5 neurons. In contrast, in future upper-layer callosal-projecting neurons *Ctip2* is repressed by de-acetylation of H4K12 at its promoter region via NuRD complex and HDAC1 recruitment by the DNA-binding protein SATB2 [314–316]. After fate specification in the early postnatal period, HDAC1 is gradually removed from the *Ctip2* promoter by the transcription factor LMO4, leading to re-establishment of H4K12ac and consequentially re-expression of *Ctip2* in a subset of upper-layer neurons [317, 318].

Several other mouse models presented with deficits of callosal projections. For example in *Cbp* knockout mice (Rubinstein–Taybi Syndrome) a reduced size of the corpus callosum was found [319], similar to the phenotype of mutants for the chromatin remodelling complex members *Prdm8* and *HP1γ* [320, 321]. Furthermore, knockdown of the chromatin remodeler *Chd8* in the neocortex impaired dendrite and axonal growth and branching of upper-layer callosal projection neurons, and resulted in delayed migration of cortical neurons at E18.5, as the majority of labelled cells remained in the VZ/SVZ [322]. Moreover, mutations in the ISWI complex member *Smarca5* cause partial agenesis of the corpus callosum, specifically due to reduced generation of viable upper-layer pyramidal neurons during mid-neurogenesis [271]. Lastly, a mouse model for *Arid1b* and *Smarcb1* deficits (Coffin-Siris Syndrome) indeed mirror the human phenotype, as *Arid1b*^+/−^ and *Smarcb1*^+*/inv*^ *NesCre*^+*/−*^ mice also have a significantly reduced corpus callosum thickness [277, 282]. Brain-specific *Smarcb1*^+/−^ mice showed agenesis of the corpus callosum due to midline glia defects, similar to human CSS patients with mutations in *SMARCB1, SMARCE1* and *ARID1B* [277]. In a human patient cohort with non-syndromic callosal abnormalities, mutations in *ARID1B* were found to be the most common cause, accounting for 10% of all cases [323].

During the axonal crossover, a multitude of axon guidance factors are required, and defects in the expression of those factors can also cause callosal projection deficits. A recent study described that chromatin remodelling of the axon guidance cue *Sema6a* caused the callosal defects observed in WAGR Syndrome (OMIM# 194072), a complex disorder including aniridia, kidney tumours, genital abnormalities, and ID [324]. Specifically, this study identified a novel protein, C11orf46/ADP ribosylation factor like GTPase 14 effector protein (ARL14EP), of which mutations were previously associated with ID [325]. C11orf46 is a member of the SETDB1-KRAB associated protein (KAP1)-MCAF1 chromatin repressor complex, and controls H3K9 methylation levels at the *Sema6a* promoter, cell-autonomously in projection neurons [324]. The callosal projection phenotype could be rescued by targeted H3K9 re-methylation at the *Sema6a* locus, indicating a direct epigenetic repressive control over axon guidance receptors in callosal-projecting neurons.

### Epigenetic modulation in GABAergic neuron development

Besides glutamatergic excitatory neurons, the mammalian neocortex contains between 12 and 20% GABAergic inhibitory neurons [326, 327]. Defects in GABAergic neurons feature prominently in NDDs, in for example epilepsy, Schizophrenia, and ASD (reviewed in [328]). Broadly, GABAergic neurons can be subdivided according to the expression of marker genes Parvalbumin (PV +), Somatostatin (SST +), and Serotonin Receptor 3a (5-HT3aR +) [329, 330]. Within each of those three large groups, further subdivisions can be made according to gene expression [331, 332], morphology, and electrophysiological firing parameters [333, 334], with current estimates ranging from 20 to 60 subdivisions [335].

The mechanism of GABAergic neuron generation is comparatively well-conserved between mice and humans [336, 337]. In mice, GABAergic neurons are generated in subdivisions of the Ganglionic Eminences (GEs: Medial GE (MGE), Lateral GE (LGE), Caudal GE (CGE), and Preoptic Area (POA)), which are temporary proliferative zones at the site of the future Striatum. In contrast to the developing cortical plate, the precursors in the GE do form a SVZ, but are not all anchored to the basal membrane, and this population is massively expanded in the human GEs [336]. The precursors divide asymmetrically to produce future GABAergic cells, which gather in the mantle zone and migrate in two morphogen-directed streams towards the cortical plate [338, 339]. The MGE and POA express the transcription factor *Nkx2-1* and produce the majority of PV + , and SST + neurons [338, 340] (Fig. 2). In contrast, VIP + neurons (the largest subset of 5-HT3aR + neurons) are produced in the CGE (see for reviews: [338, 341, 342]). Although the networks of transcription factors that define cellular identities during GABAergic neuron development and migration are comparatively well-researched [341], data on epigenetic regulation and especially chromatin remodelers is scarce and mostly has been inferred from other cell types including cancer biology and other neuron classes [343].

The first evidence for involvement of chromatin remodelers in GABAergic neuron production was reported in mice with a knockout for the histone acetyltransferase *Kat6b/Querkopf,* where a reduced density of GAD67 + (GABAergic) neurons in the cortex was found [344]. Years after the initial mouse study, mutations in human *KAT6B* were found to cause Ohdo/SBBYS syndrome (OMIM# 603736) [345, 346], however the initial findings regarding GABAergic neuron density have not been followed up to date. Mutations in the related *KAT6A* histone acetyltransferase were also found to cause ID and craniofacial dysmorphism [347, 348], recently described as KAT6A Syndrome [349]. Mouse studies have reproduced a craniofacial phenotype via *Hox* gene regulation [350], however neurodevelopmental phenotypes have not yet been studied. We do have a more complete picture for the ATP-dependent chromatin remodeler CHD2, which in humans is associated with epilepsy and broad-spectrum NDDs as described above [351]. Specifically, *Chd2* transcription is found to be activated in MGE/POA progenitors by the transcription factor NKX2-1, and by doing so the CHD2 protein in turn colocalizes with NKX2-1 on its downstream targets, illustrating the feedback loops in which chromatin remodelers act [352]. *Chd2*^+/−^ mice display a marked reduction in MGE-derived GABAergic neuron production, which results in a reduced PV + /SST + GABAergic neuron count in the cortex [202]. The functional consequences (defects in inhibitory synaptic transmission, altered excitatory/inhibitory balance, and behavioural abnormalities) were rescued by an embryonal transplantation of MGE-derived GABAergic neurons, which indicates that already a reduced GABAergic neuron production can produce profound circuit abnormalities [202].

Haploinsufficiency of the epigenetic regulator *ARID1B,* which we previously discussed as the causal gene for Coffin-Siris Syndrome, was found to cause premature apoptosis in MGE precursors in mice. As a result, *Arid1b*^+/−^ mice show a reduced production of MGE-derived (PV + and SST +) GABAergic neurons, and altered laminar arrangement of PV + and SST + neurons in the cortex (Fig. 2) [353]. Mechanistically, the same study found a general reduction of the permissive histone mark H3K9ac3 at the *Pvalb* promoter in *Arid1b*^+/−^ mice, resulting in reduced PV transcription throughout development into the adult cortex [353]. Conditional *Arid1b* knockout in specific GABAergic neuron population showed an interesting bifurcation of effects, as PV-specific *Arid1b* haploinsufficiency led to reduced mobility and social deficits, while SST-specific *Arid1b* haploinsufficiency led stereotyped behaviour such as excessive grooming [354].

Also mutations in the histone acetyltransferase CBP (Rubinstein–Taybi Syndrome), have been implicated in GABAergic precursor generation [355]. Constitutive heterozygous *Cbp* knockout mice show a transient impairment in GABAergic neuron formation in vivo [356]. Using a more direct approach, region-specific *Cbp* knockout in the developing MGE reduces the number of PV + and SST + neurons in the cortex and results in a prominent seizure phenotype [357], indicating that epigenetic regulation by CBP is directly required for proper cell-type specification of inhibitory GABAergic neurons. These results were also confirmed outside of the cortex in non-cortical areas, as conditional knockout of CBP in cerebellar progenitors lead to cerebellar hypoplasia and altered morphology of the cerebellum in both mice (*hGFAPCre::Crebbp*^Fl/Fl^ P25) [358] and patients [359]. On a cellular level, conditional knockout of CBP in granule cell progenitors altered cerebellar foliation as a result of loss of glial endfeet on the pial surface by Bergmann glia fibers and abnormal Purkinje cells arborisation [358].

After the formation of GABAergic precursors, the immature GABAergic neurons migrate tangentially following two morphogen-directed streams along the developing cortical plate, where they subsequently invade the cortex in the late stages of corticogenesis between E19 and P4 [341, 360, 361]. The exact place and time for programming the subdivisions within PV + , SST + and 5-HT3aR + is an area of active debate, with different hypotheses highlighting programming at the progenitor stage, during migration to the cortex, or only by local factors in the cortical plate. A recent study indicates that for MGE-derived neurons, the subtype is determined prior to migration, and instructs the migratory route and the place of integration in the cortex [362]. Specifically, the SST + subgroup of Martinotti cells and the PV + subgroup of translaminar PV + neurons preferentially migrate through the Marginal Zone, along the outer side of the developing cortical plate [362]. Migrating GABAergic neurons sense a multitude of environmental cues and integrate them to gene expression patterns. Similarly to GABAergic neuron progenitors, the cascades of transcription factors in migrating GABAergic neurons are comparatively well-characterized, but epigenetic modulations have only recently come into focus (see for review [343]). A recent series of studies investigated cortical GABAergic neurons derived from the POA, which produces subgroups of SST + , PV + and Reelin + GABAergic neurons [341]. Specifically, POA-derived GABAergic neurons suppress the expression of the transcription factor *Pak6* during migration via a non-canonical recruitment of the PRC (specifically EZH2) by DNMT1 to the *Pak6* promoter [363, 364]. In POA-specific *Dnmt1*-knockout mice, the repressive mark H3K27me3 is reduced around the *Pak6* transcription start site, leading to precocious expression of *Pak6* during migration and consequentially precocious activation of a post-migratory genetic program. As a result, a large portion of POA-derived neurons undergo apoptosis before reaching their cortical destination in POA-specific *Dnmt1*-knockout mice [363].

After migrating to the cortex, GABAergic neurons distribute throughout the layers, in a cell-type and area-specific pattern. Broadly speaking, PV + and SST + neurons predominate in the mid- to lower layers, whereas 5-HT3aR + are predominant in layer 1 [365] and (the VIP + subset) in layer 2/3 [366]. Primary sensory areas contain a higher density of PV + neurons, while the areas at the edge of the cortical plate contain a higher density of SST + neurons [366]. Once the GABAergic precursors are located at the appropriate cortical area and laminar location, they integrate into the local circuitry as it develops [367, 368]. The SST + GABAergic neurons mature relatively early, around the same time as the excitatory neurons in the same circuit [362, 369]. However, PV + GABAergic neurons mature much slower, and are dependent on external inputs that activate the local circuit for a successful maturation. The activity levels need to be translated to gene expression patterns, and while no complete mechanism is currently known, the high number of PV + neuron maturation dysfunctions caused by mutations in chromatin remodelers is indicative of a tight epigenetic control over this process [370]. One example is the maturation of PV + neurons in *MeCP2*^+/−^ mice, which is the primary cause for Rett Syndrome (OMIM# 312750) in humans [371–375]. It is characterized by arrested development between 6 and 18 months of age, regression of acquired skills, loss of speech, stereotypic movements (classically of the hands), microcephaly, seizures, and mental retardation. In *MeCP2*^+/−^ mice, the lack of MECP2 leads to a premature maturation of PV + cells including marker expression, morphology, and synaptic properties [372, 376]. MECP2 directly binds to the promoter regions of *Pvalb* and *Gad1*, the rate-limiting GABA synthesizing enzyme [377, 378]. Also at the adult level, neuronal activity regulates the expression of PV in a dynamic manner [379], a phenotype which is also impaired in PV + neurons of *MeCP2*^+/−^ mice [380], indicating an epigenetic component to the integration of the activity-dependent signal. In contrast, haploinsufficiency of the histone methyltransferase *Ehmt1* (Kleefstra Syndrome) causes delayed maturation of PV + neurons in the mouse sensory cortices, consisting of delayed PV expression and PNN generation, as well as reduced GABAergic neurotransmission [381]. Besides neuronal activity levels, PV + neurons also integrate morphogenic signals such as the released transcription factor *Otx2*. OTX2 is not produced in the cortex, but rather released by thalamic afferents and the Choroid Plexus [382–385]. OTX2 is taken up by the future PV + neurons, where it upregulates the expression of *Gadd45b/g*, two DNA demethylases which then mediate the up/downregulation of large sets of genes necessary for the maturation to full PV + cells, including *Pvalb* itself [382].

### Epigenetic modulation during glia development and function

At the end of the neurogenic period, cortical RGCs cells switch to glial production and generate a vast number of astrocytes and oligodendrocytes [133]. In the mouse cortex, astrocytes are first detected around E16 and oligodendrocytes around birth; however, the vast majority of both cell types are produced during the first month of postnatal development. Cre-loxP lineage tracing showed that oligodendrocytes in the cerebral cortex are produced at different sites outside of the cortex depending on the developmental stage [386]. The first wave of production begins around E12.5 in the MGE and anterior entopeduncular area. The second wave begins around E15 from in the LGE and CGE, and finally, local production begins in the cortical SVZ around birth (Fig. 2) [387]. Similar to oligodendrocytes, astrocytes can be both generated from dividing RGCs [388], from the postnatal SVZ [389], or locally by self-amplification in the postnatal cortex [118] (for detailed information about the origin and specification of glia see [390–393]). Glia play crucial roles in CNS homeostasis [394], including synaptic glutamate uptake [395], synaptogenesis [396], maintenance of extracellular potassium [397], nutrient support for neurons [398], the formation of ECM molecules [399, 400] and many other processes.

Studies investigating the role of chromatin remodelling in mouse models of NDDs have primarily focussed on the alteration of neuronal network function. Recent advances in our understanding of astrocyte function have led to the emerging concept that primary astrocyte dysfunction alone is sufficient to drive the complex behavioural phenotypes observed in some cases of NDDs. As described earlier, RGCs undergo chromatin remodelling in response to various extracellular cues to enable the accessibility of neurogenic or gliogenic genes. Loss of the H3K9 methylase *Setdb1* in mice has been shown to reduce H3K9me3 occupancy at the *Gfap* promotor, resulting in enhanced astrogenesis and accelerated differentiation (Fig. 2) [147]. Furthermore, EHMT1 has been shown to play a role in DNMT1-mediated DNA methylation via UHRF1/LIG1 interaction [401], which implies that astrocytes might contribute to the neuronal phenotypes in SETDB1-associated disorders or Kleefstra syndrome. Both SETDB1 and EHMT1 have recently been described to coexist in the same complex together with EHMT2 and SUV39H1 [402], revealing the interesting hypothesis that this complex plays an important role in the neurogenic-to-gliogenic switch, and any dysfunction in any of these genes will lead to a convergent phenotypic outcome.

Epigenetic regulation by the Polycomb Repressor Complex (PRC) has also been described to play a role in the differentiation from NPCs to astrocytes and oligodendrocytes. Acute deletion of the PRC1 component *Ring1B* or *Ezh2* at E12.5 in mice prolonged the neurogenic phase and delayed the astrogenic phase in cultures of neocortical NPCs [136]. In contrast, another report found that cerebral specific loss of *Ezh2* in the *Emx1*-*Cre* mice accelerated gliogenesis and glial differentiation at P0 [129]. Furthermore, overexpression of *Ezh2* in postmitotic astrocytes in turn lead to a downregulation of pro-astrocytic genes *S100b* and *GFAP*, whereas an increase in progenitor like genes like *SOX2* and *CD133* was found [403]. Similarly, a small population of specialized neurogenic astrocytes that resides in the SVZ and survives into adulthood expresses *Ezh2*, which is required for those astrocytes to keep their neurogenic potential [404]. These results indicate that the PRC associated proteins are essential for promoting the onset of the astrocytic differentiation of NPCs during neocortical development.

Mature glia function has been studied widely in models of NDD (including Noonan syndrome [405], Neurofibromatosis-1 [406], Costello syndrome [399, 407], Cardiofaciocutaneous syndrome [408], Fragile X syndrome [409], Alexander disease [410], and Tuberous Sclerosis Complex [411]) however only in few models of deficient chromatin remodelling. One example is a mouse model for *MeCP2* deficiency. Aside from the clear neuronal phenotype found in these mouse models, co-culture studies showed that secreted factors by *Mecp2*^*−/−*^ mouse astrocytes significantly affect the development of wild type hippocampal neurons in a non-cell autonomous manner, as was visualised by altered dendrite morphology [412]. Furthermore, neuronal phenotypes found in co-culture with *Mecp2*^*−/−*^ astrocytes appear to be dependent upon the expression of astroglial gap-junction protein Connexin-43 (Cx-43), as blocking Cx-43 restored this phenotype [413]. *Mecp2*^*−/−*^ mouse astrocytes also showed an increased expression of astroglial marker genes *Gfap* and *S100β* and abnormal glutamate clearance [414]. Interestingly, selective restoration of MECP2 in astrocytes in vivo using the Cre-loxP recombination system significantly improves locomotion and anxiety levels, and restores respiratory abnormalities to a normal pattern [415]. At the cellular level, re-expressed MECP2 in astrocytes also restores normal neuronal dendritic morphology [415]. Similar to these findings, an increased expression of GFAP and CX-43 proteins was found in the superior frontal cortex in a cohort of ASD patients [416]. Furthermore, increased levels of H3K9me3 occupancy at the promotor of the gap junction proteins Cx-30 and Cx-43 have been found in cortical and subcortical regions of patients with MDD [417]. This cohort consisted of patients expressing extremely low levels of pro-astrocytic genes *GFAP, ALDH1L1, SOX9, GLUL, SCL1A3, GJA1,* and *GJB6* [418], proposing a possible role for the H3K9 methylases SETDB1 and SUV39H1 in mature astrocyte function and CX-43 expression [417].

CHD8 is another example of a chromatin remodeler that plays a role in glia function. Recent studies show cell-type specific *Chd8* deletion in OPCs results in myelination defects in mice [243]. In addition to altered myelination, conditional knockout of *Chd8* in OPCs (*Olig1-Cre;Chd8*^fl/fl^) has been shown to slow down action potential propagation as a result of impaired myelination, leading to deficits including increased social interaction and anxiety-like behaviour as similar to *Chd8* heterozygous mutant mice [244] and behavioural phenotypes found in patients.

Heterozygous loss of *Smarca4* (*Brg1*^*fl/fl*^, *Nestin-cre*) was furthermore shown to cause precocious neuronal differentiation before the onset of gliogenesis [419]. This resulted in a significant reduction of astrocyte and oligodendrocyte differentiation in these animals, suggesting *Smarca4* controls the switch from neurogenesis to gliogenesis [419]. Furthermore, SMARCA4 is known as a mediator of long-range interactions of enhancer regions and TTSs [420], and by doing so is involved in the STAT3 dependent [421] inter-chromosomal gene clustering of *Gfap* and *Osmr* resulting in transcriptional enhancement of these genes [422]. Interestingly, loss of SMARCA2 in *SMARCA2*^K755R/+^, and *SMARCA2*^R1159Q/+^ NPCs resulted in a reduction of *Smarca4* mRNA expression, together with an increased and functionally active binding to chromatin [423]. These results suggested that mutations in *SMARCA2* result in global retargeting of *SMARCA4*, which was shown to drive de novo activation of enhancers and upregulation of astrocyte genes [423].

To summarize, current evidence shows that chromatin remodelers play a role in the development, migration and circuit integration of each of the major cortical cell classes: Glutamatergic and GABAergic neurons and glia. Consequentially, failures of chromatin remodelling can impact the development of each of those cell types, resulting in a lasting impairment in cellular function.

## Future perspectives

In this review, we detailed the contribution of chromatin remodelers in different neural cell-classes during the multiple stages of the developmental continuum. Chromatin remodelers are crucial parts of a cell’s information processing machinery, by integrating external and internal signals into gene expression patterns. Developing neurons inhabit an extraordinarily complex epigenetic landscape, and events such as cell-type specification are under tight epigenetic control [424]. Consequentially, defects in chromatin remodelling will lead to a relaxation of that epigenetic control, causing for example premature neural differentiation at the expense of progenitor pool expansion [208].

As chromatin remodelers have such a variety of functions in different cell types, timepoints and at specific genetic loci, a full picture requires concurrent measurements at several levels simultaneously—a task that current technologies are only starting to address. We see the potential for progress in the following fields:

1. Understanding chromatin remodeler locus specificity: Chromatin modifications are site-specific on the genome level, such as histone methylation at the activity-dependent *Bdnf* exon IV [425]. However, until recently, to study this site-specific targeting one had to rely on the cell’s innate targeting abilities. Coupling catalytic subunits to a precise targeting protein allows artificial induction of locus-specific chromatin modifications. One example is the dCas9-SunTAG method [143, 426], where a genetic locus is tagged via dCas9 and gRNAs. Subsequently, local chromatin is modified by a chromatin modifier’s catalytic subunit targeted towards the tag. The ability to induce chromatin modifications at specific genomic sites will improve our understanding of the regulatory networks in gene expression, for example during cell fate specification.
2. Understanding the role of chromatin remodeler presence in complexes: Chromatin modifiers exist in complexes that dynamically assemble, disassemble, and bind to chromatin at different locations. Complexes are hypothesized to differ between different locations (or time points), however those have proven difficult to investigate with classic immunolabelling techniques. Recent advances in spatial proximity labelling, such as promiscuous biotinylation targeted via dCas9 [427], allow for a precise snapshot of protein complexes assembled in spatial proximity to a single genomic region. Importantly, this technique can be applied in living cells and in vivo in the developing brain [428], making it applicable to the neurodevelopmental questions that we have detailed here. This technique allows detailed insights site-specific complex dynamics, a largely unexplored feature of the genetic landscape.
3. Chromatin remodelling temporal specificity: Neuronal specification is thought to be a series of tightly controlled gene expression (and hence epigenetic regulation) states. For glutamatergic neuron generation in the cortex, recent evidence points to a stochastic generation of different subtypes [121, 429], however it is currently unknown whether GABAergic neuron generation is controlled in a similar way [361]. Classic labelling techniques such as BrdU were only able to identify neurons born within approximately 12 h from each other, which is slower than the hypothesized changes in genetic expression state. Recently developed labelling techniques such as FlashTag selectively label neurons born in a 2-h window in vivo, leading to a more precise identification of the transcriptional program controlling glutamatergic neuron specification [424, 430]. Application of the same technique for GABAergic neurons might deliver interesting insights into subtype specification as well.
4. Measuring cell-type specificity: The classification of the brain’s cells has been controversial since the start of neuroscience as a field. For example, GABAergic neurons and glia have long resisted simple classification [431, 432]. However, recent large-scale single-cell RNA sequencing studies [331, 332, 433–435] attempt to map the cellular diversity of brain from the bottom up. Furthermore, studies measuring multiple modalities on the same neurons promise a unification of classifications from single-cell electrophysiology, morphology and RNA sequencing (Patch-Seq), and have delivered insights in glutamatergic [436] and GABAergic neuron populations [333]. Especially when coupled with advanced analysis techniques [334], those large datasets might soon be available as a “reference classifier” that experimental data can be compared with, similarly to reference atlases in neuroanatomy or reference genomes in genomics.
5. Identification of converging molecular pathways for therapeutic interventions: Functional interactions between several NDD related chromatin remodelers and their regulatory proteins has been shown to converge on a shared transcriptional axis [156, 287, 437–439]. One example is the H3K4 demethylase KDM5C, whose expression is controlled by three regulatory proteins: ARX, ZNF711 and PHF8. All four of those genes are located on the X chromosome, and consequentially mutations in any of these four genes are associated with X-linked NDDs [440–442]. Interestingly, loss of *Arx* caused a significant reduction of *Kdm5c* expression and neuronal maturation in *C*. *Elegans* and mice, which could be restored using the HDAC inhibitor SAHA [438]. These findings imply that chromatin remodelers function in closely coupled transcriptional networks, with mutations in genes in the same cluster producing overlapping NDD phenotypes [439, 443]. As demonstrated in the case of *KDM5C*, overlapping regulatory pathways might be used as drug targets, and mutations in shared pathways could prove to be relatively easily identifiable biomarkers [444, 445]. In a promising first step, several groups have shown that chemical inhibition of HDACs can successfully rescue behavioural phenotypes in mouse models of NDDs [116, 438, 444–447]. Furthermore, the fact that those pathways tend to be well-conserved might prove valuable in translation to clinical practice.

The studies summarized in this review were almost exclusively performed in animal models of NDDs. While mice have many advantages as model organisms and many features are conserved down to the cellular level [331], some features appear to be unique to the human lineage, for example specialized cell-types such as outer radial glia cells [448], subpial interlaminar astrocytes [449], or the recently described rosehip neurons [450]. Furthermore, some time periods in neurodevelopment are much longer in comparison, elongating the vulnerable periods for many regulatory processes in humans. Therefore, using mouse brains as the sole model we might overlook important human-specific aspects of brain development and NDD pathogenesis. Although the use of animal models will remain essential to study complex developmental processes like cortical layering or migration, human induced pluripotent stem cells (hiPSCs) offer a higher-throughput model to investigate the developmental continuum from the earliest point of progenitor specification until the formation of neuronal circuits in vitro. For this reason, the use hiPSCs has gained a lot of attention recent years in the field of NDD research. HiPSCs provide an unlimited pool of (patient) material, which can be differentiated into neuronal networks, and can be monitored over development in vitro. In addition, these cells are comparatively easy to manipulate using for example CRISPR-Cas9 genome editing, and therefore can be used as a high throughput tool to study genotype–phenotype correlations in a controlled environment [451, 452]. Moreover, patient specific hiPSCs carry the same genetic background as the patient, which allows the study of polygenic disorders like ASD or Schizophrenia that cannot be modelled using animal models.

Protocols for the differentiation of hiPSCs into 3D cerebral organoids are becoming increasingly popular as these models have been shown to resemble the complex developmental programs of early corticogenesis during the first and second trimester of human foetal development [453, 454]. Indeed, 3D cerebral organoids derived from patients with severe microcephaly as a result of *CDK5RAP2* mutations showed reduced neuroepithelial differentiation, fewer and smaller progenitor regions, and premature neuronal differentiation [455]. Furthermore, 3D human organoids from idiopathic ASD patients showed reduced proliferation of progenitors, increased neurogenesis, and an imbalance between the production of glutamatergic and GABAergic neurons [456]. Moreover, organoids derived from *CNTNAP2*^+/−^ hiPSCs showed increased organoid volumes as a result of increased proliferation of progenitors, which in turn expanded the neuronal population [457]. Recent work has shown that patient-derived iPSC organoids with copy number variants in the ASD risk locus 16p11.2 mirror the patient’s micro/macrocephaly phenotype [458]. Similarly, *RAB39b* loss in 3D organoids has recently been shown to cause hyperproliferation and enlarged organoid size [459]. Studies are currently exploring organoid vascularization to further extend the development and complexity of these organoids [460–462], which will allow in the future to study more complex brain phenotypes using these in vitro approaches.

In summary, despite lots of progress in the field, the full influence of chromatin remodelling on neurodevelopment is currently unknown. To fully understand chromatin remodelers’ influence throughout the developmental continuum and identify possible human-specific pathways, future studies should combine human-specific in vitro models such as 3D cerebral organoids and well-characterized developmental models such as mice.

## Data Availability

Correspondence and should be addressed to n.nadif@donders.ru.nl.
